# Cryo-EM resolves the structure of the archaeal dsDNA virus HFTV1 from head to tail

**DOI:** 10.1126/sciadv.adx1178

**Published:** 2025-10-03

**Authors:** Daniel X. Zhang, Michail N. Isupov, Rebecca M. Davies, Sabine Schwarzer, Mathew McLaren, William S. Stuart, Vicki A. M. Gold, Hanna M. Oksanen, Tessa E. F. Quax, Bertram Daum

**Affiliations:** ^1^ Living Systems Institute, University of Exeter, Exeter EX4 4QD, UK.; ^2^ School of Natural Sciences, Faculty of Environment, Science and Economy, University of Exeter, Exeter EX4 4QD, UK.; ^3^ Henry Wellcome Building for Biocatalysis, Biosciences, Faculty of Health and Life Sciences, University of Exeter, Exeter EX4 4QD, UK.; ^4^ Department of Molecular Microbiology, Groningen Biomolecular Sciences & Biotechnology Institute, Faculty for Science and Engineering, University of Groningen, 9747 AG Groningen, Netherlands.; ^5^ Faculty of Health and Life Sciences, University of Exeter, Exeter EX4 4QD, UK.; ^6^ Molecular and Integrative Biosciences Research Programme, Faculty of Biology and Environmental Sciences, University of Helsinki, Viikinkaari 9, 00014 Helsinki, Finland.

## Abstract

While archaeal viruses show a stunning diversity of morphologies, many bear a notable resemblance to tailed bacterial phages. This raises fundamental questions: Do all tailed viruses share a common origin and do they infect their hosts in similar ways? Answering these questions requires high-resolution structural insights, yet no complete atomic models of archaeal viruses have been available. Here, we present the near-atomic resolution structure of Haloferax tailed virus 1 (HFTV1), an archaeal virus thriving in extreme salinity. Using cryo–electron microscopy, we resolve the architecture and assembly of all structural proteins and capture conformational transitions associated with DNA ejection. Our data reveal genome spooling within the capsid and identify putative receptor-binding and catalytic sites for host recognition and infection. These findings uncover key mechanisms of archaeal virus assembly, principles of virus-host interactions, and evolutionary links connecting archaeal, bacterial, and eukaryotic viruses.

## Teaser

This structure of an archaeal tailed virus (arTV) provides detailed insights into arTV assembly and infection mechanisms.

## INTRODUCTION

Viruses outnumber their hosts by at least a factor of 10, making them the most abundant biological entities on Earth. Viruses infecting microorganisms are diverse and those specific to archaea encompass an astonishing variety of morphotypes, such as spiral, bottle, and spindle-shaped viruses (*1*). Of all different morphotypes, viruses with a helical tail and an icosahedral head containing a double-stranded DNA (dsDNA) genome are extremely successful. Tailed viruses (TVs) are the dominant archetype of bacterial viruses, infecting all major bacterial lineages, driving cellular evolution, and shaping ecosystems, in particular in the oceans, where they play a crucial role in nutrient cycling (*2*–*5*).

Archaea are also infected by a diverse group of TVs (arTVs), which are evolutionarily related to the bacteria-infecting TVs (baTVs), and have been grouped together with them in the class *Caudoviricetes* (*6*, *7*), in which several new arTV-specific families have been established. Specifically, halophilic and methanogenic archaea belonging to the *Euryarchaea* are often infected by arTVs and encode arTV-like proviruses (*7*–*16*). In addition, arTV-like proviruses are found in the genomes of other major classes of archaea, such as *Thaumarchaea*, *Aigarchaea*, and *Thermoplasmata* (*17*–*20*).

Comparisons of arTV and baTV genomes suggest an ancient divergence, and arTVs represent an evolutionarily distinct group within the prokaryotic dsDNA virome (*6*). The evolutionary link between the two groups is suggested by their common architecture and conserved proteins, especially their major capsid proteins (MCPs) (*21*, *22*). On the basis of the broad distribution of TVs, they are hypothesized to be a part of the virome of the last universal common ancestor (*6*, *23*).

The structures and infection mechanisms of dsDNA TVs have been studied predominantly for bacterial virus members of the *Caudoviricetes*. Like baTVs, arTVs also have different tail types, which group them into podo-, myo-, and siphovirus morphotypes (*7*). In general, the tails are involved in host recognition, binding, penetration of the cell envelope, and genome delivery into the host cell (*24*, *25*). Some structural proteins of baTVs also have enzymatic activity, such as the peptidoglycan hydrolase activity of the tape measure proteins (TMPs) of mycobacteriophages (*26*). However, the lack of full atomic structures for TMPs precludes a comprehensive understanding of their function.

After genome delivery, the transcriptional program of the virus is activated. The viral genome is replicated and progeny particles assembled in the cytoplasm. The assembly of the head is initiated here during virus maturation (*27*). The usually icosahedral, but sometimes prolate heads are assembled from MCPs, guided by scaffolding proteins to obtain the correct capsid geometry. These scaffolding proteins are degraded once the heads are fully assembled (*27*). A portal protein (PP) is inserted into the capsid’s head, through which the viral genome is loaded. After genome packing, the PP acts as the anchor point for the tail (*27*).

The viral genome is packaged into the empty head in an energy-dependent manner by a terminase complex transiently docked to the portal vertex. Previous cryo–electron microscopy (cryo-EM) studies of baTVs have shown that dsDNA typically forms coaxial spools within viral capsids, in which the DNA coils into shells around an axis aligned with the portal (*28*–*30*). These shells display increasing disorder toward the center, possibly due to the inner layers being further from the capsid and therefore less influenced by the icosahedral structure.

While other types of genome spooling such as concentric and toroidal organizations have been suggested, the coaxial spooling model is the most widely accepted model for dsDNA inside icosahedral capsids (*30*). However, molecular dynamics studies reproducing physical properties and packaging forces of the capsid have suggested a more disordered organization, often with multiple configurations (*31*). These models have also revealed that the organization of the DNA depends on the size and shape of the capsid; in simulations, prolate icosahedrons display toroidal packing, compared to coaxial spooling in isometric icosahedrons (*30*). After DNA packaging, the portal is plugged and connected to the tail. The short-tailed podoviruses are assembled directly on the portal vertex, while the longer tails of myo- and siphoviruses are first assembled and connected later to the head (*27*).

The infection and assembly mechanisms of arTVs are less studied than those of baTVs. However, it was recently shown that the receptor for two arTVs is the S-layer protein (*32*, *33*), which is the main cell envelope component of archaea. We have demonstrated that the siphovirus *Haloferax* TV 1 (HFTV1) adsorbs to its host *Haloferax gibbonsii* LR2-5 via its tail, but also with its head (*13*). HFTV1 is hypothesized to bind the cell first reversibly by its head and then irreversibly via its tail, before ejecting its DNA into the host cell (*33*). However, because of a lack of structural information, the functional significance of this unusual binding mechanism remained elusive.

Comparative genomics has indicated that arTVs have MCPs with the same Hong Kong 97 (HK97) fold as those conserved amongst baTVs and eukaryotic herpesvirus (*34*). By electron cryo-tomography (cryo-ET) it was shown that the head of the arTV *Haloarcula sinaiiensis* TV 1 (HSTV-1) consists of multiple MCPs with a HK97 fold, evolutionarily linking the arTVs with baTVs and herpesviruses (the realm *Dublodnaviria*) (*21*, *22*, *35*). However, the complete structure of any arTV virion has not yet been resolved, and it is unclear to what extent arTVs compare with baTVs.

Here, we purified HFTV1 virions and studied their structure via cryo-EM. Through single-particle analysis, we obtained near-atomic resolution maps of DNA-filled and empty virions, enabling us to assemble full atomic models of the virus pre- and post-DNA release, including all structural protein components. Comparing both structures enabled us to propose a model that describes the hitherto elusive host-cell adsorption and infection mechanism. In addition, we were able to visualize the packaged dsDNA, revealing how the DNA is spooled inside the capsid.

Our structure aids in tracing the evolutionary history and structural diversity TVs and provides fresh insights into the common principles that underpin the global success of these widely distributed prokaryotic viruses.

## RESULTS

### Structures of the HFTV1 virion pre and post DNA ejection

Virus particles were purified, plunge-frozen on holey carbon grids, imaged using a Titan Krios TEM, and reconstructed using RELION-4.0 and 5.0 (*36*, *37*). During image processing, we noticed that the sample contained DNA-filled particles as well as those that had already ejected their DNA genomes. These were easily distinguishable during 2D classification (figs. S1 and S2), allowing for the two classes of particles to be processed separately and thus viral structures pre and post ejection to be compared.

**Fig. 1.**
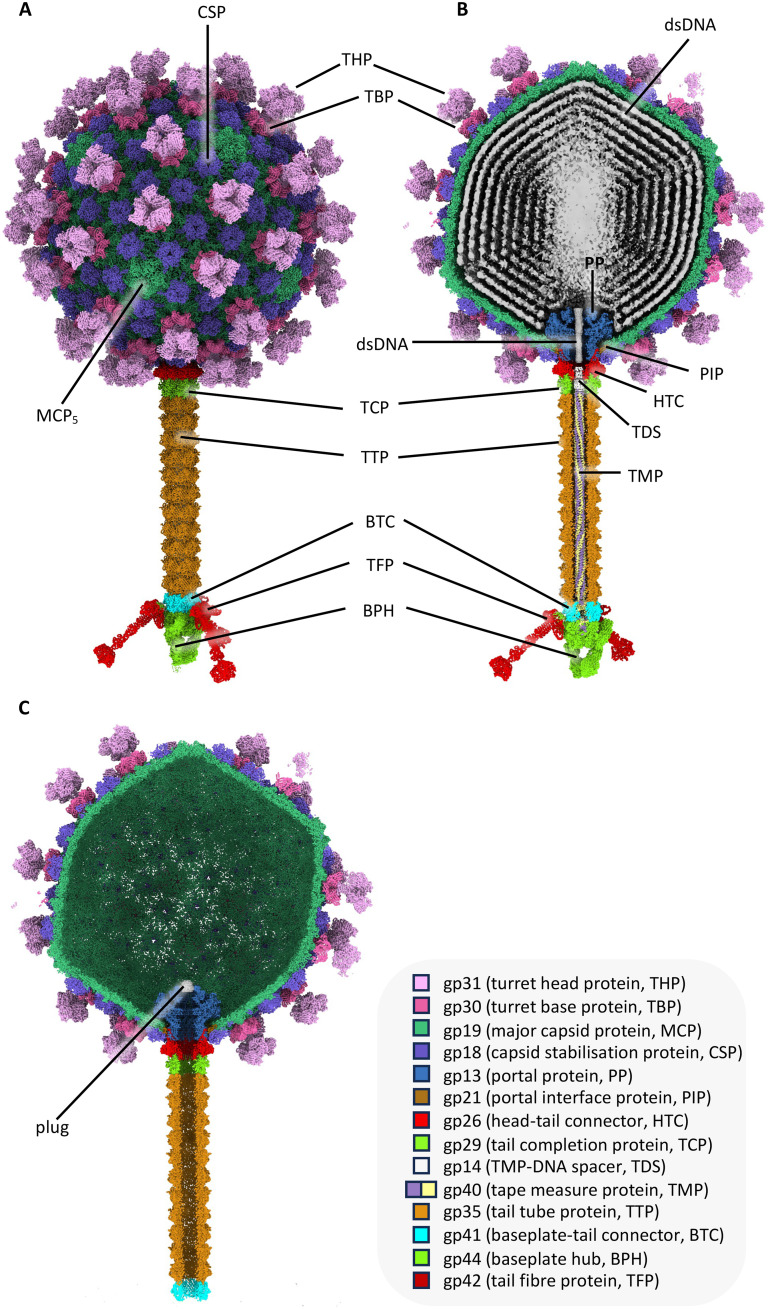
Overall structure of HFTV1. (**A** and **B**) Structure of head-tailed and turreted archaeal virus HFTV1 containing dsDNA in side view (A) and cross section (B). (**C**) Cross section of the empty virus, where the dsDNA, tape measure protein, baseplate hub and tail fibers are missing. Instead, a plug-like density appears to cap the portal protein. The empty structure corresponds to a postinfection state.

The final map of the whole infectious DNA-filled virion had a global resolution of 4.39 Å (flowchart S1; fig. S3). To obtain higher resolutions of its constituent parts, the head, turrets, portal, tail, baseplate, and tail fibers were subjected to focused refinements using symmetry operators pertaining to each substructure (flowchart S1; figs. S4 and S5). The combined data allowed for atomic model building of the entire virion, aided by ModelAngelo (*38*) (movie S1). The full atomic structure (Fig. 1, A and B) revealed that the virus has a length of 1350 Å and an icosahedral head with a diameter of ~600 Å. The head is decorated by up to 60 turrets, which project ~90 Å above the surface of the capsid and thus increase its diameter to ~780 Å. The tail has a length of 560 Å and terminates into a baseplate with three radiating TFs of ~190 Å in length.

Compared to its DNA-filled counterpart, the empty virus lacks the tape measure protein (TMP), baseplate hub (BPH), and tail fibers (TFs), indicating that these parts are jettisoned during DNA ejection (Fig. 1, B and C). However, the length of the tail tube remains unaltered, demonstrating that it does not contract during DNA ejection, similar to siphoviruses that infect bacteria (*39*, *40*). Moreover, the heads of filled and empty viruses show no notable differences, indicating that DNA release does not lead to any structural changes in the head.

Structural interpretation and atomic model building were informed by proteomic analysis (see Materials and Methods), as well as AlphaFold2 (*41*) predictions. Mass spectrometry analysis of the infectious virions was performed, which led to the identification of several structural proteins (data S1). Only one protein band showed an exact match with the predicted amino acid sequence of the protein gp29 (GenBank accession QAS68862.1). gp21 and gp14 were not detected, but the hypothetical proteins gp20, gp17, and gp43 were identified. Full peptide data are provided in data S1.

AlphaFold2 was used to predict the structures of each of the 68 proteins encoded by the genome of HFTV1 (*13*), both as monomers and various multimers where hardware capacity allowed. This expedited the assignment and refinement of confidently annotated sequences and allowed candidate proteins with no identified proteomic counterpart to be shortlisted.

### The head has right-handed *T* = 7 icosahedral symmetry and is studded with turrets

**Fig. 2.**
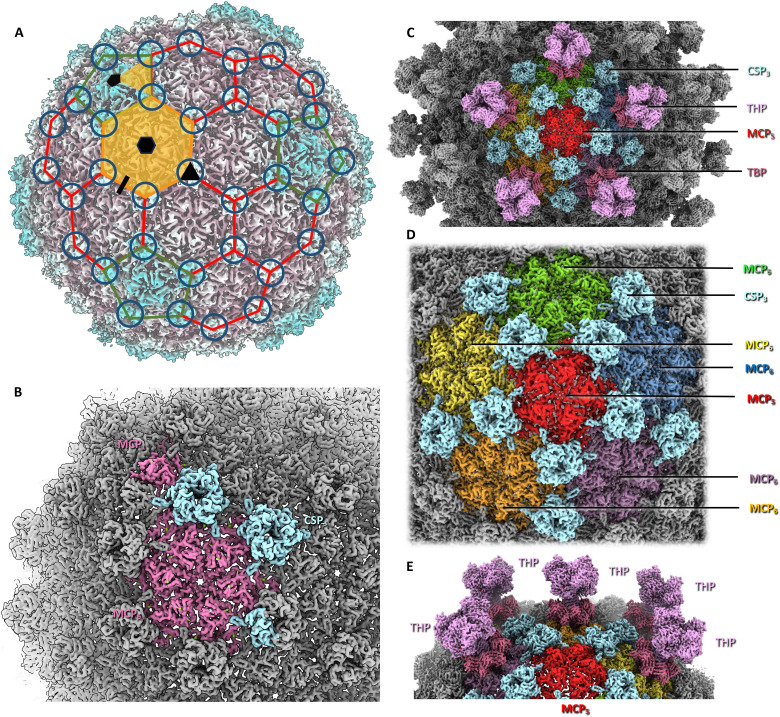
Structure of the icosahedral head of HFTV1. (**A**) icosahedral capsid with major capsid protein hexamers (MCP_6_) outlined in red and MCP pentamers (MCP_5_) outlined in green. The asymmetric subunit is highlighted in orange. Hexameric, pentameric, trimeric, and dimeric interfaces between MCPs are shown as black hexagon, pentagon, triangle, and line, respectively. The black pentagon, triangle, and line also demarcate five-, three-, and two-fold axes of icosahedral symmetry, respectively. The location of capsid stabilization proteins (CSPs) trimers is indicated by circles. The background map is colored by radius in ChimeraX. (**B**) Close-up of the asymmetric subunit. MCP in pink, CSP in light blue. In (A) and (B) turrets have been omitted for simplicity. (**C**) Cropped view of the head, centered on an MCP_5_. The locations of the CSPs and turrets consisting of the turret base protein (TBP) and turret head protein (THP) are indicated. (**D**) Close-up of a central MCP_5_, surrounded by 5 MCP_6_ and 10 CSP trimers (CSP_3_). Turrets have been omitted for clarity. (**E**) Diagonal view of MCP_5_, showing that each MCP_6_ carries a turret, while all MCP_5_ remain unoccupied.

Icosahedral symmetric averaging of the viral capsid produced a 2.36 Å map (flowchart S1, fig. S4), which was used to build an atomic model. The capsid forms a right-handed (dextro) icosahedron of a triangulation number *T* = 7 [*h* = 2, *k* = 1; (*42*, *43*)], made up of 60 copies of the seven-protein asymmetric repeating unit (Figs. 1 and 2). *T* = 7 icosahedral quasi-symmetry is characteristic of several bacterial siphoviruses (*44*, *45*). However, the handedness of HFTV1’s capsid is unusual, as most resolved structures of both tailed and untailed *T* = 7 icosahedral capsids display left-handed (levo) symmetry (*22*, *46*–*51*). To date, only a handful of viruses have been shown to form *T* = 7 dextro capsids via structural analysis, including three tailed bacteriophages (*52*–*54*) and the untailed eukaryotic papovaviruses (*55*).

The base layer of the capsid consists of gp19, the MCP. The MCP forms alternating pentamers and hexamers, which interact through asymmetric trimers (Fig. 2A). In each trimer, the MCPs adopt three distinct conformations. Around the portal, the conformation of MCP is again different from those found in the trimers, to accommodate the capsid-portal interface. Across the capsid, 13 different MCP conformations were found (fig. S6A and movie S2).

DALI (*56*) revealed that HFTV1 MCP is structurally similar to MCPs of tailed bacteriophages. The top hits were MCPs of Mycobacterium phage Che8 [PDB-8E16; (*57*); *Z* score, 18.7], Klebsiella phage Kp9 (PDB-7Y23; *Z* score, 18.8), and bacteriophage HRP29 (PDB-8ELD; *Z* score, 18.4). The MCP of HFTV1 is missing the N-terminal amino acids 1 to 100 compared to the protein sequence predicted from the genome (fig. S6B), suggesting that the protein is post-translationally processed, possibly during maturation. Lacking the first 100 amino acids, HFTV1 MCP fits the classical HK97 fold. The interior-facing surfaces of the MCP are highly negatively charged, which has been suggested to prevent the packaged DNA from sticking to the capsid, thus facilitating rapid release upon infection (*58*).

The trimeric MCP interfaces are bound by trimers of gp18, the capsid stabilization protein (CSP; Fig. 2). Across the icosahedral capsid, these CSP trimers are arranged in a series of hexagonal and pentagonal rings, which coincide with the faces and vertices of the icosahedron, respectively (Figs. 1A and 2). HFTV1 CSP binds the outer surface of each MCP trimer, thus stabilizing the capsid architecture. According to DALI (*56*), HFTV1 CSP has potential homology with capsid proteins from tailed bacteriophages, such as the head fiber gp8.5 N base of bacteriophage phi29 [PDB-6QYY; (*59*); *Z* score, 13.1], the CSP of marine siphovirus TW1 [PDB-5WK1; (*51*); *Z* score, 11.8], and the Cyanophage Pam3 MCP [PDB-8HDT; (*60*); *Z* score, 11.8].

One Mg^2+^ ion is coordinated at each MCP-CSP interface (fig. S7A). Each subunit in the MCP hexamer contains two intermolecular Mg^2+^ ions, as well as coordinating two additional Mg^2+^ ions in each subunit-subunit interface. In the MCP pentamer, each subunit instead coordinates three intermolecular Mg^2+^ ions and one in each interface. In addition, each CSP trimer binds a K^+^ ion in the central cavity (fig. S7B). The extensive intra- and intermolecular metal ion coordination suggests a stabilizing role of these divalent ions.

### The dsDNA packaging in the head is revealed

**Fig. 3.**
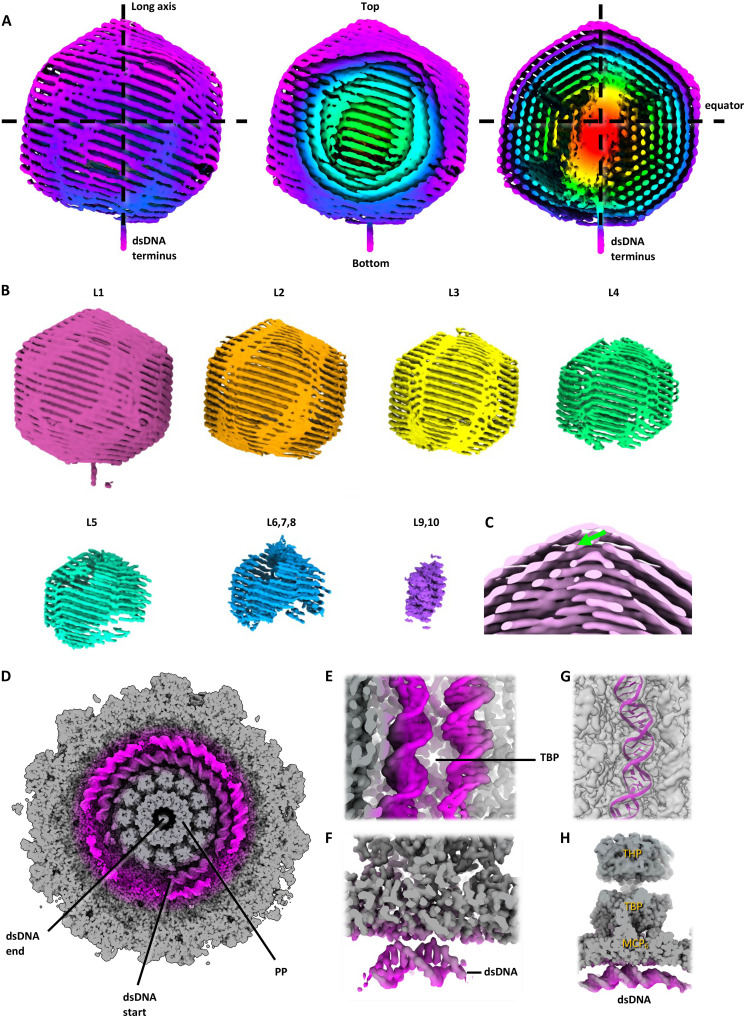
dsDNA spooling inside the virion head. (**A**) Cryo-EM map of the 10-layered dsDNA spool in full view (left), with the first four layers cropped (middle), and cropped through the center of the dsDNA (right). The dsDNA emerges from the bottom of the spool at the portal. Note that the spooling direction of the DNA is inclined with respect to the equatorial plane of the capsid. (**B**) The layers (L) of the dsDNA shown separately. The outer layers L1-L3 are resolved best, while layers L4-L10 show an increasing proportion of unresolved areas, indicating disorder. (**C**) Close-up of the spool at the opposite end to the portal. The green arrowhead indicates dsDNA descending from layer 1 into layer 2. (**D**) View of the portal area of the capsid (PP, portal protein). Layer 1 of the dsDNA (magenta) is particularly well resolved around the portal and the twist is clearly visible. The putative terminus of the DNA molecule is indicated. (**E** to **H**) Various views of two parallel dsDNA strands of layer 1 beneath an MCP hexamer (H). In this region, base pairs are resolved [(E) and (F)] allowing a section of dsDNA to be modeled (G).

To elucidate the organization of dsDNA inside the capsid, we isolated dsDNA density from our cryo-EM map by subtracting protein signal from an asymmetric capsid reconstruction, followed by 3D refinements using CryoSPARC (*61*) (Fig. 3A, flowchart S2, and fig. S8). To enhance resolution in the innermost DNA layers and in regions near the portal and the opposite capsid pole, we performed focused particle subtraction and refined these areas separately (flowchart S2, fig. S8, and Fig. 3B).

Our analysis revealed that the dsDNA is arranged into 10 concentric shells (Fig. 3B). We observed two termini of the dsDNA: One extends through the portal (Fig. 3A), while the other is likely located in the outermost shell, encircling the portal’s circumference (Fig. 3D). dsDNA near the portal is better resolved compared to other capsid regions, especially at the hexagonal capsid faces adjacent to portal-proximal turrets (Fig. 3, D to H). These hexagonal vertices may serve as anchoring points, facilitating dsDNA packing coordination. The twist and base pairs of the dsDNA are clearly visible (Fig. 3, D to F), conforming to canonical B-DNA structure (Fig. 3G). This contrasts with the A-form DNA reported in viruses infecting hyperthermophilic archaea, such as *Sulfolobus islandicus* rod-shaped virus 2 (SIRV2) (*62*–*65*).

Similar to other dsDNA viruses like siphoviruses and herpesviruses (*66*, *67*), HFTV1 shows tightly packed DNA layers, with higher resolution in outer layers and around the portal. The central capsid region exhibits denser DNA packing than the outer layers, possibly indicating a specialized zone or presence of cargo proteins. Masked refinement confirms that this dense central region primarily consists of DNA, specifically the two innermost layers (layers 9 and 10) packed more tightly than the outer eight (Fig. 3B).

Overall, HFTV1 displays left-handed DNA spooling, akin to that seen in herpes simplex virus 1 (HSV1) (*66*) and *Bacteroides intestinalis* virus ΦcrAss001 (*68*). From the portal-adjacent terminus, the DNA forms spooled layers inclined at ~17° relative to the capsid “equator,” or the axis parallel to the portal base (Fig. 3A). The center-to-center spacing between dsDNA strands within and across layers 1 to 7 is approximately 25 Å, consistent with previous observations of dsRNA spooling, where in-layer spacing is ~26 Å (*69*). However, interlayer spacing in dsRNA is wider (~29 Å), indicating that dsDNA in HFTV1 is packed more tightly.

The inclination angles of the dsDNA vary slightly across layers: the outer six layers (1 to 6) exhibit inclinations of 16°, 18°, 17°, 17°, 17°, and 16°, while the resolution of layers 7 to 9 is insufficient for accurate measurements (Fig. 3, A and B). In the inner core, strand spacing decreases to 23 Å and inclination to 11° (Fig. 3, A and B). This inner region diverges from the spooled pattern, adopting a barrel-like conformation (*69*). Unlike dsRNA viruses where strands move between layers at capsid vertices (69), HFTV1 dsDNA forms depressions at these vertices without apparent interlayer transitions (Fig. 3A). The cause of these depressions is unclear, as no corresponding protein or other density was observed.

Genomic analysis of HFTV1 using InterPro (*70*), HHpred (*71*), and arCOG (*72*) identified several predicted DNA binding proteins, including various Zn-finger domain–containing proteins (datasets S2 and S3). These DNA binding proteins might mediate the observed ds-DNA packing in the capsid. However, due to resolution limits inside the dsDNA-packed environment, no such DNA binding proteins resolved.   Thus, although auxiliary packaging proteins may contribute, the mechanism driving the dsDNA packaging transition and its transition between layers is still unknown. Clarifying the precise roles of these DNA binding proteins therefore requires further experimentation.

Although dsDNA and dsRNA viruses differ evolutionarily and use distinct packaging mechanisms, the convergent evolution of spooling likely confers biological advantages, possibly facilitating genome ejection during infection. Both must mitigate curvature stress and electrostatic repulsion within tightly packed genomes. Comparative structural analyses may reveal how these physical constraints are managed.

While most dsDNA strands in HFTV1 can be traced, pockets of disorder remain, particularly near the poles of the spool. Because of the DNA’s inclination, these disordered regions do not coincide with capsid poles but rather with adjacent triangular faces. The disordered pockets may contain DNA loops formed as strands approach the poles and spooling angles increase. Such loops could relieve packing stress, similar to those observed in HK97 bacteriophage packaging simulations by Coshic *et al.* (*31*). However, those simulations show large interlocking loops rather than the coaxial spooled layers seen here. These differences may stem from the absence of a packaging motor in the simulations. In addition, there is currently no structure for a packaging motor for archaeal dsDNA viruses, which could provide deeper insights. It is possible that a distinct threading mechanism is employed by the packaging motor of archaeal dsDNA viruses, leading to layered spooling rather than interlocking loops, though this remains speculative.

Apparent gaps can also be seen near the dsDNA's terminus near the portal, even though the dsDNA in this area is otherwise well resolved (Fig. 3D). These gaps may either also correspond to disordered loops or variations in the exact position of the terminus among virions. The increased disorder seen at the DNA’s top pole (distal from the portal) likely reflects heterogeneous DNA descent paths into inner layers that were averaged together during classification (Fig. 3C).

On the basis of our data, we propose the following DNA spooling path in HFTV1: Starting from the terminus encircling the portal at the bottom pole, DNA winds upward to form the outermost shell. At the pole opposite the portal, DNA descends into the second layer (Fig. 3C), then spools back down toward the portal pole, reversing direction again. This spooling repeats nine times until the DNA reaches the central core and exits through the portal.

### The turrets harbor a conserved deacetylase-like domain

**Fig. 4.**
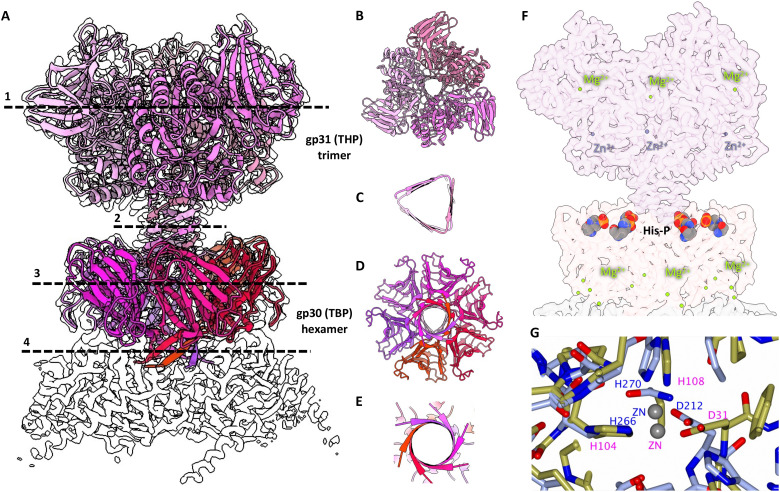
Structure of the turret. (**A**) Side view of the turret, consisting of a turret head protein (THP) trimer and a turret base protein (TBP) hexamer. The underlying MCP hexamer is shown in white. Cross sections through THP (1), the THP stalk (2), the TBP (3), and the TBP stalk (4) are indicated by dashed lines and shown 90° rotated in (**B** to **E**). (**F**) Transparent map of the turret (side view) with coordinated Mg^2+^ (green) and Zn^2+^ (purple), as well as putative phosphohistidine residues (multicolor) indicated. (**G**) Superposition between the proposed active site of HFTV1 THP (blue) and that of the polysaccharide deacetylase from *M. smegmatis* MC2 155 (PDB-3RXZ; yellow).

Each hexagonal MCP of HFTV1 forms the base of a turret. In icosahedral and asymmetric reconstructions of the capsid, the turret heads were poorly resolved, an indication of flexibility. To improve the resolution of the turret map, localized reconstruction techniques were used (flowchart S1). Particles were symmetry-expanded around the fivefold-symmetric head-tail axis, sampling the five particularly well-defined turret complexes adjacent to the portal. Threefold rotational (C3) averaging focused on a single turret position produced a 2.36-Å-resolution map (flowchart S1). Turrets were found to consist of two separate proteins; a base formed of a hexamer of gp30 [turret base protein (TBP); Fig. 4], and a trimeric turret head protein (THP) gp31 (Fig. 4). Notably, the TBP density contained more amino acids than suggested by the previously annotated genome (fig. S9).

Both the TBP hexamer (Fig. 4, A and D) and THP trimer (Fig. 4, A and B) have mushroom-like architectures (Fig. 4A). The TBP inserts into the MCP hexamer with a short stalk formed by a six-stranded β-barrel (Fig. 4E). Extensive Mg^2+^ ion coordination involving two ions per TBP subunit stabilizes the interaction between TBP and MCP (Fig. 4F and fig. S7A). The THP trimer then inserts into the TBP hexamer with a longer, 12-stranded triangular barrel (Fig. 4C), elevating the THP above the TBP. Six histidine modifications (likely phosphohistidines) are found at the hexamer-trimer interface (Fig. 4F). While all these modified histidines belong to the TBP, three directly interact with the N termini of the THP trimer via salt bridges, contributing to the stability of the interaction between the THP and TBP.

Alongside three Mg^2+^ ions, the THP trimer includes three catalytic Zn^2+^ coordination sites (Figs. 4, F and G, and S7D). Analyzing the structure of the THP using DALI (*56*) and National Center for Biotechnology Information (NCBI) Basic Local Alignment Search Tool (BLAST) (*73*) indicated a similarity between THP and polysaccharide deacetylases from multiple species (data S4). Superimposing the structure of the THP with that of the polysaccharide deacetylase from *Mycobacterium smegmatis* (PDB-3RXZ) results in a close match between the catalytic domains of both proteins (fig. S10).

InterProScan (*74*) reports that the deacetylase-like domain encompasses residues 199 to 413, ending at the C terminus of the THP. However, a second conserved domain is also reported, corresponding to a galactose-binding domain-like superfamily (G3DSA:2.60.120.260), which spans residues 57 to 198 and makes up the second externally facing subunit of the THP.

We found that the turrets are assembled in two possible orientations, where the turret head trimer can be rotated by 60° with respect to the turret base hexamer. This rotational flexibility may increase the chance of binding between turrets and the S-layer of *H. gibbonsii*.

### The portal and its integration into the capsid

**Fig. 5.**
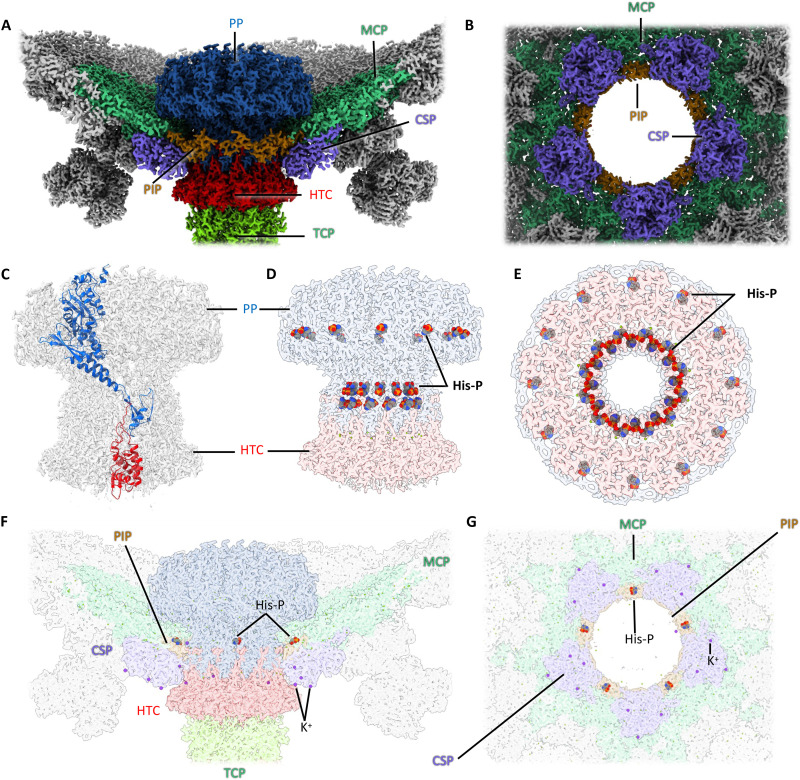
The portal. (**A**) Cross section through the portal region with the portal protein (PP; gp13, blue) in the center. A pentameric ring of gp21 (portal interface protein, PIP, brown) integrates the portal 12-mer into the fivefold vertex of the capsid. (**B**) Top view of the portal interface, the PP has been omitted for clarity. (**C**) Cryo-EM map of the portal in transparent gray, with PP (blue) and head-tail connector (HTC; gp26, red) monomers shown as atomic models in ribbon representation. (**D** and **E**) Cryo-EM map of the portal in side (D) and top (E) views. PP in transparent blue and HTC in transparent red. The PP 12-mer contains 36 putative phosphohistidine residues (His-P; red and blue spheres). Twenty four of these phosphohistidines form a ring lining the portal tunnel. (**F** and **G**) Transparent views of (A) and (B), respectively. Putative phosphohistidine in the portal-capsid interface, as well as coordinated K^+^ (purple dots) are indicated.

The portal is a dodecamer of gp13 (PP; Fig. 5, A and C), which spans the capsid through one pentagonal vertex (Fig. 5B). We found that the symmetry mismatch between the MCP pentamer and the PP dodecamer is interfaced by the intermediary portal interface protein gp21 (PIP; Fig. 5, A and B). Modeling PIP into an asymmetric reconstruction of the capsid-portal interface showed that its annotated genome sequence was eight residues too short, suggesting that the starting methionine is different from that originally annotated (fig. S11). At the portal-capsid interface, one of the MCP-bound magnesium ions is replaced by an arginine of PIP. In addition, PIP also has a putative phosphohistidine residue at position 17, which forms a stabilising H-bond with neighboring MCP monomer (Fig. 5, F and G).

**Fig. 6.**
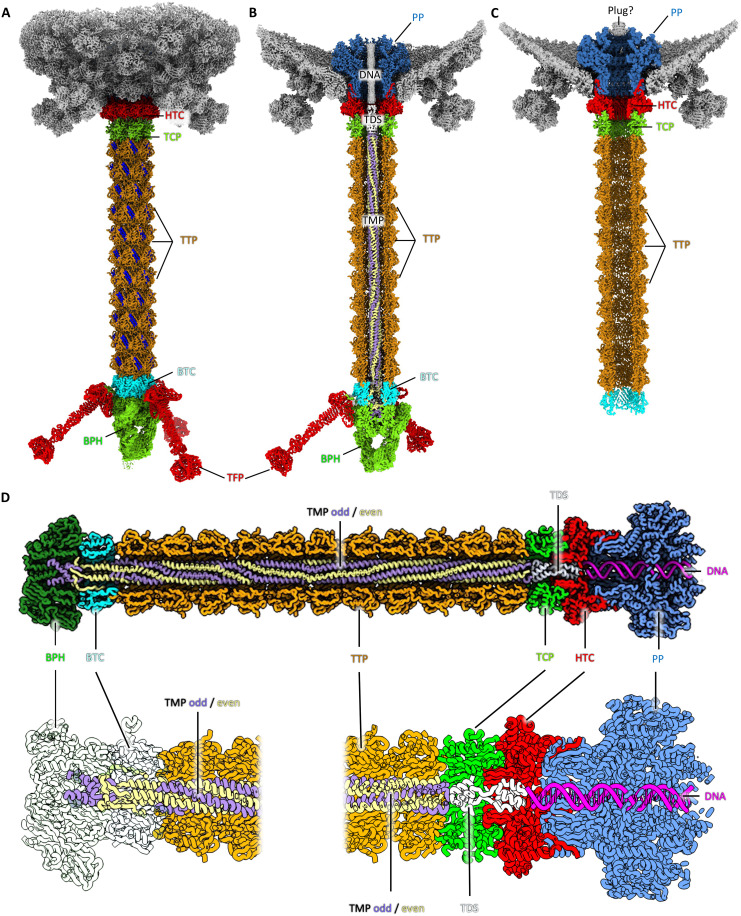
The tail. (**A**) Full structure of the tail consisting of a dodecameric head-tail connector (HTC), a hexameric tail completion protein (TCP), 11 tail tube (TTP) hexamers, a trimeric baseplate-tail connector (BTC), a trimeric baseplate hub (BPH), and three trimeric tail fibers (TFs). The same pair of β-strands per TTP monomer is highlighted in blue, showing the 23.6° rotational offset between the TTP hexamers. (**B**) Cross section of the tail tube, revealing a hexameric tape measure protein (TMP), projecting from a cavity in the BPH to a vestibule formed at the TCP. The TMP and dsDNA are separated by a spacer (TDS), and the dsDNA traverses through the portal into the capsid. (**C**) Structure of the tail of the empty virus. The dsDNA, TMP, and baseplate are missing. Instead, a density appears to plug the capsid-facing side of the portal. Otherwise, the portal and the components of the tail tube are unchanged in width, length, and conformation, indicating a rigid structure. (**D**) Close-ups of cross sections through the atomic model of the DNA-loaded tail.

Twelvefold rotational averaging produced a 2.34-Å-resolution map of the PP (Fig. 5A and flowchart S1). In the mature virion, the terminus of the dsDNA protrudes through the central channel of the PP (Fig. 6, B and C), which is lined with a ring of putative phosphohistidine residues (Fig. 5, D and E). These residues would add negative charge to the portal tunnel and likely disfavor any DNA binding to the portal, thus facilitating rapid DNA release during infection. We do not observe the positively charged rings that have previously been reported for other PPs and have been implicated in preventing DNA-backsliding during translocation or the stabilization of DNA within the capsid before tail attachment (*75*–*78*). The absence of these rings would suggest that a different backstop mechanism is implemented in HFTV1.

Comparing the portal structures of the DNA-filled and empty capsid shows that the latter has a plug-like density, which is missing in the former (Fig. 6, B and C). The structure of the PP itself does not show substantial differences between the two states, indicating that large scale conformational changes in this protein are not required to facilitate DNA release.

### A complete structure of the tail and TMP

Particles recentered on the tail underwent refinement applying C3 symmetry, which resolved the tail at 2.45 Å resolution (Fig. 6, A and B, fig. S5). The tail region begins with dodecameric gp26 [head-tail connector (HTC)], which interacts with the PP to maintain a conduit through which DNA can pass. This is followed by hexameric gp29 [tail completion protein (TCP)], and subsequently, 11 copies of hexameric gp35 [tail tube protein (TTP)] making up the bulk of the tail (Fig. 6, A, B, and D). Each hexameric segment of the tail is rotationally offset from the next by 23.6° (Fig. 6A).

The tail forms a hollow tunnel of 36 Å in diameter. In the DNA-filled virus, this tunnel houses gp40 (TMP), a hexameric coiled-coil structure with alternating C-terminal conformations. The TMP extends from the central channel of the first TTP near the portal to a cavity in gp44 [baseplate hub (BPH)] (Fig. 6, B and D). On the basis of our map, we could build the TMP as a full atomic model, with all side chains present. The C terminus of the TMP (residues 317 to 341) is well-defined in the density, with odd-numbered subunits forming α-helical structures docked into the BPH (Fig. 6D), while the even-numbered subunits form β-stands structure folding back toward the N terminus (Fig. 6D). For both, odd and even TMP subunits, residues 2 to 316 fold into α-helical structures. These bundle into a hexameric coiled-coil with threefold symmetry, spanning most of the tail length. In the extended N-terminal part of the protein, the density remained ambiguous in various regions, likely due to flexibility, and side chains were poorly resolved. The coiled-coil region of the TMP precisely aligns with the 11 tail tube rings, confirming that its length dictates tail tube length. As the coiled coil unravels at the C-terminal region, it likely guides the addition of tail-terminating proteins. Specifically, the three folded-back TMP termini interact with the trimeric baseplate-tail connector (BTC; gp41), while the three extended termini engage the trimeric BPH protein. This unraveling thus also facilitates the transition from hexameric TMP symmetry to the trimeric BTC and BPH (Fig. 6D).

An elongated density within the channel of PP clearly corresponds to dsDNA (Fig. 6B). In addition, we observe two densities between the DNA and the TMP, located within the TCP and HTC proteins (Fig. 6B). Using AlphaFold3 (*79*), we predicted the structures of oligomers for all short HFTV1 proteins of unknown function, resulting in a dimer of gp14 as the best match for this density. The gp14 dimer forms two globular domains, one at the N, and the other at the C terminus, connected by short loop regions (Fig. 6D). Because of the strong features of threefold symmetry of the TMP, we were unable to align particles based on the weaker features of gp14 and the neighboring DNA, leading to an averaging out of secondary structure details of this region. However, superimposing the dimer of gp14 as a rigid body resulted in a reasonable fit. Because of its location between the TMP and DNA, we name this protein the TMP-DNA spacer (TDS). In the empty virus, the TMP and TDS are absent (Fig. 6C), demonstrating that they are released during DNA ejection.

### The BPH forms an asymmetric trimer carrying three TFs

**Fig. 7.**
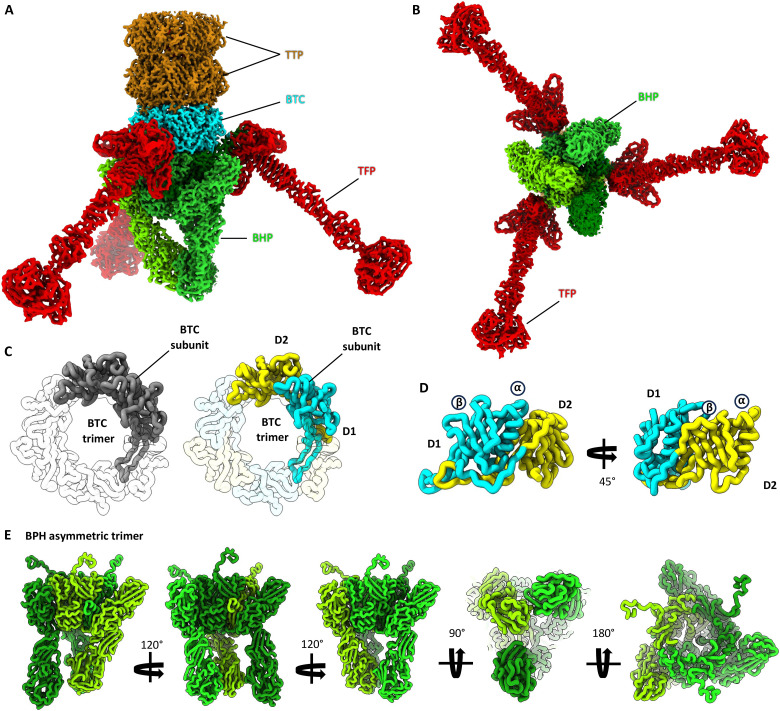
The baseplate. (**A** and **B**) Structure of the HFTV1 baseplate in side (A) and end-on view (B). (**C**) Left, BTC trimer in top view with one of the subunits colored in gray and the other two transparent; right, the same subunit is colored by domain (D1, cyan; and D2, yellow). (**D**) Two side views of the BTC subunit, rotated by 45°, showing that both domains adopt the same fold. Both domains consist of a β-sheet followed by an α-helix. (**E**) Various views of the asymmetric baseplate hub (BPH) protein trimer (monomers colored with different shades of green). Three extended domains project downward from the core of the BPH. Each of the domains has a different conformation. While two interact directly, the third is unbound and thus more flexible.

The final TTP hexamer is followed by the trimeric BTC, which in turn interfaces with the trimeric BPH (Fig. 7, A and B). Notably, the BTC protein is a pseudo-hexameric trimer: Each subunit consists of two homologous domains, which have likely arisen through a gene duplication and fusion event (Fig. 7, C and D). The BPH coordinates Mg^2+^ and K^+^ ions that likely contribute to the stability of the complex, and connects to the BTC via a triangular ring, from which three globular domains project (Fig. 7, A and E). Curiously, these domains show an asymmetric configuration (Fig. 7, A, B, and E). While two of the domains are bound to each other, one remains unbound. As the binding interfaces of these three domains are equal, it can be assumed that they are free to interchange (movie S3). This suggests that the three BPH domains may undergo dynamic cycles of binding and unbinding, which in turn may aid virus’ docking or penetration of the S-layer.

The BPH also anchors three TFs (Fig. 7, A and B). The TFs are connected to the N terminus of each BPH subunit and radiate away at a ~45°. This interaction is stabilized by extensive hydrogen bonds and Mg^2+^ ion coordination. A DynaMight (*80*) analysis revealed that the TFs are highly flexible with respect to the BPH, likely aiding their ability to dock to the S-layer at the cell surface (movie S4).

The TFs are formed as a homotrimer of gp42 [TF protein (TFP)]. Three TFPs come together to form a base, followed by a β-helix, a stalk, and a head. The TFPs coordinate Mg^2+^ and Zn^2+^ ions (fig. S7C), which likely stabilize the trimer interface. According to DALI (*56*), the BPH shows some structural similarity with xylanases and glycosaminidases (data S4). For the TFPs, DALI suggests some similarity with the CspB protease from *Clostridium perfringens* [PDB-4I0W; (*81*); *Z* score, 8.5], albeit the *Z* score is very low. This may hint at the BPH and TFPs playing a role in binding to the surface glycans of *H. gibbonsii* or even the degradation of the S-layer. However, further experimentation will be required to confirm this.

### Coordinated magnesium ions are crucial for virion stability

To test whether the multiple intra- and intermolecular Mg^2+^ ions coordinated throughout the BPH, TFPs, turrets, and HTC proteins (fig. S7A) have a stabilizing role, we depleted Mg^2+^ from infectious virus particles using chelating agents. This resulted in a loss of infectivity (fig. S12) and fragmentation of the virus particles (fig. S13), showing that Mg^2+^ coordination aids structural integrity. The extensive ion binding within the capsid is likely a result of HFTV1 evolving in a highly saline environment, where Mg^2+^ ions are abundant.

### Conservation and divergence of HFTV1 compared to archaeal and bacterial TVs

Although HFTV1 infects archaea, its overall architecture is strikingly reminiscent of bacterial siphoviruses. To investigate whether HFTV1 is related to bacteriophages, we performed a structural similarity analysis for each component protein of HFTV1 using the DALI server (*56*). Probable homology (*Z* scores between 8 and 20) with proteins of bacteriophages was determined for the majority of HFTV1 structural proteins. These include (in descending order of *Z* score) MCP, THP, TCP, CSP, TTP, BTC, and TFP (data S4).

DALI suggested that the PP and the BPH of HFTV1 are definite homologs of phage counterparts (with *Z* scores of >20). The greatest *Z* scores were identified in Thermus phage G20c portal (PDB-4ZJN; *Z* score, 20.2) and prophage MuSo2 43-kDa tail protein (PDB-3CDD; *Z* score, 20.1), respectively.

Similarity, but no definite homology, could be determined for the HTC, and the PIP had no hits for structural similarity whatsoever, suggesting that this part of the capsid has a unique origin. The THP also did not show any homology with bacteriophage proteins, but instead with bacterial polysaccharide deacetylases (top *Z* score of 18.6 with an enzyme from *Mycolicibacterium smegmatis* MC2 155; PDB-3RXZ). Another intriguing probable homolog was found in an endo-1,4 β-xylanase from the termite species *Trinervitermes trinervoides* [PDB-7AX7; (*82*)] with a *Z* score of 18. For the TBP, only one probable homolog could be identified, a phage-like element PsbX protein from *Bacillus subtilis* strain 168 [PDB-6IA5; (*83*); *Z* score, 8.1].

These data hint at the possibility that HFTV1 is directly evolutionarily related to tailed bacteriophages and that core structural proteins, particularly those forming the portal, capsid, tail, baseplate, and TFs, could be products of horizontal gene exchange between bacterial and archaeal viruses. Other proteins that convey specific adaptations to the archaeal host, such as the THPs, appear to have evolved specifically for HFTV1.

## DISCUSSION

**Fig. 8.**
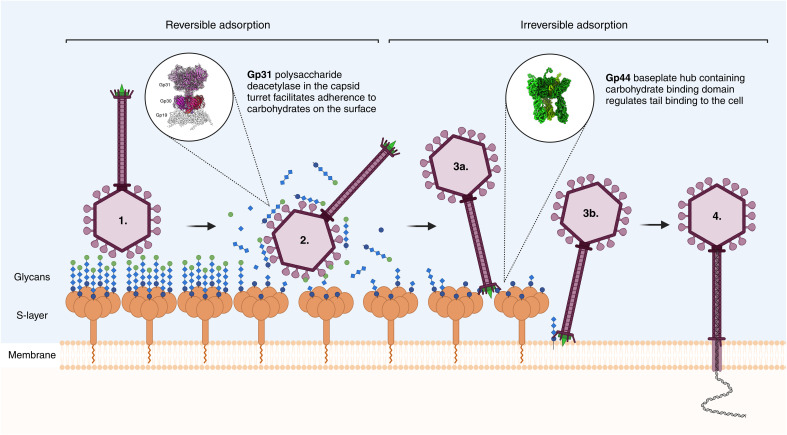
Proposed multistep receptor binding mechanism driving HFTV1 attachment to *Haloferax gibbonsii* LR2-5 and viral dsDNA delivery. (**1**) HFTV1 binds reversibly to host glycans (chains of blue and green circles and diamonds) associated with the S-layer via the N-terminal domain of the THP (gp31). (**2**) Simultaneously, the C-terminal polysaccharide deacetylase-like domain of the THP degrades the glycan matrix, weakening the S-layer of the host. (**3a**) After glycan degradation, irreversible binding is mediated by a carbohydrate-binding domain on the BPH (gp44), which presumably binds to glycan residues on the S-layer. (**3b**) Alternatively, it may bind to lipid-glycan carriers in the membrane. This secondary binding event reorients the particle. (**4**) The BPH-mediated binding induces conformational changes that facilitate genome ejection into the host cytoplasm, accompanied by the loss of the BPH and TFs.

Our structural analysis of the HFTV1 virion revealing putative glycan-binding sites, combined with previous observations of particle attachment, suggests a multistep mechanism for viral adsorption (Fig. 8). We propose that HFTV1 initially adheres reversibly to host glycans via the proposed N-terminal carbohydrate-binding module of the THP, which forms the outermost region of the turrets. Simultaneously, the C-terminal region of the THP, with its predicted glycoside deacetylase/hydrolase activity, would modify the host’s glycan matrix, potentially weakening the protective surface layer lattice of *H. gibbonsii*. It is conceivable that in this process, new binding sites are revealed that facilitate baseplate anchoring following a reorientation of the virus.

The baseplate, which serves as an anchor point on the cell membrane, secures the viral particle firmly to the host cell. This attachment by the baseplate, which is irreversible in many other viruses (*84*–*86*), reorients the virus so that the tail axis is aligned perpendicularly to the cell surface. The C-terminal end of the BPH contains a predicted carbohydrate/galactose-binding domain, which may interact with remaining glycan residues on the S-layer or lipid-glycan carriers embedded in the membrane. The potentially dynamic baseplate structure may act in a lock-and-key mechanism that opens or disrupts the interaction between S-layer proteins, creating a pathway for virus genome delivery. Trimeric baseplates with carbohydrate-binding domains have also been reported for bacteriophages (*87*).

The tail has a length of 560 Å, sufficient to span the distance between the S-layer and the cell membrane, which is ~200 Å in *Haloferax volcanii* (*88*) and likely similar in *H. gibbonsii*. This enables the virus to penetrate the S-layer and the membrane. After the disassembly of the BPH and tail fibers, a conduit from viral portal complex into the host cell is formed, through which the viral genome is then injected.

Some phages have peptidoglycan-hydrolyzing activity in their TMPs to degrade bacterial envelope components (*26*, *89*). Proteolytic domains are not evident in the TMP of HFTV1, suggesting that the virus may not use this proteolysis mechanism to overcome the S-layer. Instead, the tail may penetrate the envelope mechanically, akin to a needle. Genome release in HFTV1 triggers structural changes in the distal tail region, including BPH detachment and TF dissociation. These findings suggest that the baseplate and TFs not only anchor the virus but also facilitate or initiate genome ejection.

To our knowledge, structural proteins with predicted carbohydrate-degrading domains have previously not been reported for archaeal viruses. However, carbohydrate-binding and/or hydrolytic proteins have been identified as components of bacteriophages (*90*–*93*). Several of these proteins are involved in hydrolysis of the bacterial peptidoglycan layer; in most cases, these carbohydrate binding/hydrolytic proteins form the tail spike, baseplate, or are part of the TFs (*90*–*93*). P22 podophage infecting *Salmonella typhimurium* has a peptidoglycan/murein hydrolase (gp4) inserted in the head structure that degrades peptidoglycan (*94*). In addition, glycan binding domains have also been found in the capsid head of myophage T4, which are involved in adhering T4 to the mucus layer of the human gut, to increase the chances of encountering a susceptible *Escherichia coli* host (*95*). Thus, multistep adsorption processes are common among bacteriophages (i.e., phage λ, T5, and SPP1). Usually, binding to cell envelope saccharides is the first reversible step that maintains and transitorily orients the phage before specific receptor recognition occurs in the second step (*96*).

In HFTV1, the carbohydrate-binding domain in the THP likely serves as a first anchoring point of the virus to the cell surface. This reversible binding step increases the chance that a successful irreversible binding (via the baseplate and TFs) takes place, which then triggers DNA ejection and hence infection. As no homologs of the THP were found in other haloarchaeal viral genomes, the described strategy could be specific to HFTV1. HFTV1 was shown to have an adsorption rate that is several orders of magnitudes faster than those known for other haloarchaeal viruses (*33*). However, since several baTVs contain glycan-binding proteins, and the archaeal cell surface is highly glycosylated, we expect that structural analysis of other haloarchaeal viruses will disclose the existence of similar glycan-binding proteins.

The spooling of the dsDNA within the virus capsid is reminiscent of that displayed in several other dsDNA viruses of eukaryotes and bacteria (*66*, *67*). The prevalence of this arrangement may arise due to this conformation being both electrostatically favorable and preventing entanglement upon injection into the host cell under extremely high pressure (*31*, *66*). However, the axis of the spooling, which is out of alignment with the portal, appears unique in comparison with other tailed dsDNA viruses, such as Epsilon15 (*67*), T7 (*97*), and HSV (*66*). While HSV does display angled spooling (at least in the first layer), the axis of the spools still appears to align with the portal (*66*).

It is possible that within other dsDNA eukaryotic viruses and phages apparently displaying linear spooling, such as HSV and Epsilon15, the averaging of different DNA conformations within the capsid may have caused the spools to appear linear, as mentioned in previous studies (*98*, *99*). Since no studies of dsDNA organization have been conducted in this much detail before, it is conceivable that the observed angled spooling is common across viruses, but further cryo-EM analysis of dsDNA viruses will be required to confirm this.

We observe areas of disorder within the spooled dsDNA, which is not unusual, especially for the innermost layers of viral genomes (*31*, *66*, *97*). This disorder may be necessary to give the genome room to expand and shrink as mutations are introduced and may also aid the unraveling of the spool as it is ejected. HFTV1 has an unusually dense packaging of central layers. The tight packing and bending at high angles displayed in this structure should be energetically unfavorable, given the negative charge and relatively rigid structure of dsDNA (*100*). The curvature stress of the DNA may be sufficient to overcome the electrostatic repulsion, as demonstrated by continuum computations for the bacteriophage T7 (*101*).

As DNA is packaged inside the capsid, the translocation rate of DNA decreases when the capsid fills near capacity (*102*). In DNA packaging simulations, decreased packaging speed has been shown to increase disorder within the DNA layers (*31*, *103*). This could suggest that disordered central layers may commonly be seen across other viruses with spooling conformations. However, in HFTV1 the innermost layers display a high degree of order, indicating the possible presence of histone-like proteins maintaining this structure. Future work aiming to increase the resolution of the packaged dsDNA will shed more light on the presence and function of such DNA binding proteins in the capsids of archaeal viruses and beyond.

In summary, the structure of HFTV1 presents a major step forward in our understanding of archaeal viruses, provides structural evidence of the strong evolutionary relationship between TVs of bacteria and archaea, and opens new research directions to answer fundamental questions in virology, such as host-cell infection and virus assembly.

## MATERIALS AND METHODS

*H. gibbonsii* LR2-5 cells and HFTV1 virions were cultured, and viruses were purified by precipitation and density ultracentrifugation in sucrose and CsCl as described previously (*13*, *33*). Samples were concentrated by differential ultracentrifugation and resuspended in 18% SW buffer (2.47 M NaCl, 89 mM MgCl_2_, 85 mM MgSO_4_, 56 mM KCl, 3 mM CaCl_2_, and 48 mM tris-HCl, pH 7.2).

### Stability tests of the virions

The purified viruses were diluted 1:1000 in the following buffers: (i) 50 mM tris-HCl (pH 7.2), (ii) 10 mM EDTA, 50 mM tris-HCl (pH 7.2), (iii) 10 mM EGTA, 50 mM tris-HCl (pH 7.2), and (iv) 18% SW (positive control), and incubated at 22°C. The number of infectious viruses was determined by plaque assay after 2 and 24 hours of incubation. To analyze the effect of magnesium depletion, the purified particles in 18% SW were collected (Beckman Coulter Airfuge rotor A95, 30 psi, 30 min, 22°C), resuspended in either (i) 200 mM EDTA and 50 mM tris-HCl (pH 7.2) or (ii) 50 mM tris-HCl (pH 7.2; control) to obtain a particle concentration of 1 mg/ml, and analyzed in a linear 10 to 40% (w/v) sucrose density gradient in 50 mM tris-HCl (pH 7.2) [Thermo Fisher Scientific rotor TH641, 35,000 rpm, 1 hour 45 min (EDTA treated)/35 min (control), 15°C]. The gradients were analyzed by absorbance profiling (A260 and A280) and collection of fractions by Piston fractionator. Protein profiles were analyzed by SDS–polyacrylamide gel electrophoresis (PAGE) and infectivity of the light scattering bands was determined by plaque assay.

### Proteomics analyses by mass spectrometry

Purified HFTV1 virions were dissociated in SDS-PAGE loading buffer [4% SDS, 0.25 M tris (pH 6.8), 0.06% bromophenol blue, 0.5 mM dithiothreitol, and 10% glycerol], boiled for 5 min at 95°C and the proteins were separated on 10 to 20% gradient polyacrylamide gels (Criterion precast gel, Bio-Rad). The virus proteins were visualized by staining with Coomassie Brilliant Blue.

SDS-PAGE analysis of the highly infectious purified HFTV1 virions of high specific infectivity 2 × 10^13^ pfu/mg protein (*33*) revealed protein bands ranging in size from 250 to 20 kDa (fig. S14). The proteins in the most prominent bands (bands 1 to 8; fig. S14) were subjected to mass spectrometric analysis as described previously (*104*) at the Interfaculty Mass Spectrometry Core Facility, University of Groningen. In brief, in-gel digestion was performed on the excised gel bands with 150 ng of trypsin. Liquid chromatography–mass spectrometry (LC-MS)–based proteomics analyses were performed using two-thirds of these digests. LC-MS raw data were processed with Spectronaut (version 17.0.221202) (Biognosys) with a user-defined database containing 68 proteins (HFTV1 proteome, downloaded from GenBank, accession number NC_062739.1), using the standard settings of the directDIA workflow, except that quantification was performed on MS1. For the quantification, the *Q* value filtering was set to the classic setting and no imputation or normalization was applied.

### Single-particle data collection

Purified virions (1.4 × 10^14^ pfu/ml) were resuspended in 18% SW buffer and loaded onto R2/2 QUANTIFOIL 200-mesh copper-carbon cryo-EM grids with graphene oxide support film. Grids were blotted using an FEI Vitrobot with force −1 for 5 to 6 s, under environmental conditions of 4°C, 100% humidity. Grids were plunge-frozen in liquid ethane, then transferred to and stored under liquid nitrogen.

Data were collected in two separate sessions at the Diamond Light Source Electron Bioimaging Centre, using 300 kV Thermo Fisher Scientific Titan Krios TEMs with Gatan K3 and Falcon 4i direct electron detectors, respectively. EPU software (Thermo Fisher Scientific) was used for automated data acquisition. The first dataset (Gatan K3) consisted of 10,236 52-frame movies. The data were collected in super-resolution mode with a virtual pixel size of 0.675 Å/px, and a total dose of 54.6 e^−^/Å^2^. The second dataset (Falcon 4i) consisted of 19,717 45-frame movies, collected in counting mode with a pixel size of 1.171 Å/px, and a total dose of 50 e^−^/Å^2^. Both collections were performed with a range of defocus values (−0.8, −1.1, −1.4, −1.7, and −2.0 μm). Cryo-EM image acquisition details are summarized in table S1.

### Single-particle data processing

Single-particle reconstruction followed the established RELION-4.0/5.0 processing pipeline (*36*, *37*). Both datasets were motion-corrected using RELION’s built-in implementation under a 5 × 5 patch scheme, and CTF estimation was performed using CTFFIND-4.1 (*105*) through the integrated interface. A binning factor of 2 was applied to the super-resolution dataset during motion correction, resulting in a pixel size of 1.35 Å/px.

Virus particles were picked automatically using a 700- to 800-Å Laplacian-of-Gaussian filter, processed by 2D classification, and subsequently submitted to Topaz (*106*) through RELION for training and repicking.

Following further rounds of 2D classification, an initial model of the capsid was produced, and aligned to RELION’s I3 symmetrical convention. Empty capsids were separately selected from these classifications and returned to Topaz for training and repicking. Filled and empty particles subsequently followed similar processing pipelines.

### Unification of the tail orientations

The initial model was used to align the particles to I3 symmetry via 3D refinement. Particles were subsequently symmetry expanded in I3 using RELION’s relion_particle_symmetry_expand function, and masked classification with no alignment focused on the capsid-tail interface lying on the fivefold (*Z*) axis was performed on the symmetry-expanded data to separate out a subset of particles sharing the same tail orientation. Particles were de-duplicated, re-extracted, and recentered to focus on this capsid-tail interface region.

### Turrets

Particles were also symmetry expanded in C5, and re-extracted and recentered to focus on the position of one of the turret complexes. Further 3D refinement under C3 symmetry produced a high-resolution structure of the turret complex.

### Portal

3D refinement in C12 symmetry of the capsid-tail interface region revealed the high-resolution structure of the portal complex. Particles were realigned to a C5 map of the capsid-tail interface, and no-alignment 3D classification of the portal under C12 symmetry differentiated misorientations of the portal 12-mer relative to the fivefold capsid axis, allowing manual reorientation of each class to produce an asymmetric reconstruction of the region’s 5/12-fold symmetry break.

### dsDNA

Particles were recentered on the center of the capsid, undergoing unbiased (C1) and I3 refinements. C1 particles of full virions were imported into CryoSPARC (*61*). Particle subtraction was used to remove the capsid to reveal the DNA density. The DNA was then classified into its dominant spooling arrangements.

To obtain the structures of the layers of DNA without interference from surrounding layers, the densities of each layer were isolated and processed separately. The SEGGER tool (*107*) in ChimeraX (*108*) was used to segment the DNA into its seven different layers, with the final two layers consisting of the sixth, seventh, and eighth layers, and the ninth and tenth layers, respectively. Each layer was imported back into CryoSPARC and converted into a mask. These masks were then used in particle subtraction to isolate each layer of DNA to locally refine. Because of the increased density of the innermost layers, the mask for refining the innermost layers went through two iterations of refinement before being used in the final refinement.

Incline angles of the spooled dsDNA were measured using the more defined strands in the equatorial parts of the spool, which are more ordered and further from the disordered regions at the poles. The angles were measured against the “equator” of the capsid, defined by a horizontal parallel to the portal base.

### Tail

Masked 3D classification of the tail stub under C6 symmetry was used to separate the two possible orientations of the C6 tail connected to the C12 HTC. One class was manually rotated to align with the other, and both classes were recombined, recentered on the midpoint of the tail, and briefly refined to maintain alignment with the symmetry axis.

For empty virions, particles underwent 3D refinement in C3 symmetry to produce a final map of the tail post–DNA ejection. In addition, the class with the greater number of particles was separately re-extracted with an expanded box size to encompass the entire virus, and particles were refined to produce an asymmetric reconstruction of an entire empty virion.

### Baseplate

DNA-filled virions were further recentered on the baseplate region at the end of the tail. Particles were refined in C3, classified in C1, and manually reoriented to align the pseudo-symmetric baseplate domain conformations. Particles centered on the baseplate also underwent symmetry expansion in C3 and extraction of one of the TF positions. These were manually rotated to align with the symmetry axis and refined under C3 symmetry. Baseplate particles were further extracted back to the midpoint of the tail and refined under C1, C3, and C6 symmetry to produce maps for modeling of the tail and TMP.

### Whole virion

C1-refined particles were recentered back to the capsid-tail interface, and the box expanded for a full asymmetric reconstruction. Because of the two distinct orientations in which the C6 tail completion and TTPs could be assembled onto the C12 HTC, with particles exhibiting ~50/50 split, and likewise for the C3 BTC and baseplate onto the C6 tail, as well as the C1 pseudosymmetric BPH trimer, this resulted in 12 distinct orientations in which the entire tail complex could be assembled onto the C5 capsid. Unmasked classification of the capsid-tail interface under C5 symmetry separated the orientations, producing particle sets of approximately equal size, which could be locally refined separately to generate consensus maps of the entire virion. 

Manual reorientation of particles was performed using a custom Python script, using the packages starfile (https://github.com/teamtomo/starfile) and mrcfile (*109*) for manipulation of RELION STAR and MRC files, respectively, as well as SciPy (*110*) for rotational calculations.

3D classifications were performed without alignment and with a high T regularization value (64 or 1024). Following initial classification of the tail orientation from the symmetry-expanded particles, 3D refinements were typically performed with either local searches of small angles (≤1.875°), or global searches only about the symmetry axis by specifying the additional arguments --sigma_tilt and --sigma_psi within the RELION interface.

Refined map resolutions were estimated using the 0.143 gold-standard Fourier Shell Correlation approach (figs. S3 to S5 and S8). The strategies for cryo-EM image processing have been summarized in flowcharts S1 and S2.

### Model building and refinement

Folded protein conformations were predicted by AlphaFold (*41*) using genome sequences submitted to NCBI GenBank (accession no. NC_062739.1). Structural similarity was assessed using DALI (*56*). Maps were postprocessed for presentation using EMReady (*111*). EM density, protein chains, and structural fitting were visualized using ChimeraX (*108*).

ModelAngelo software (*38*) was used to build protein fragments in the density. Sequences of these fragments were aligned by BLAST against annotated HFTV1 sequences to identify proteins. AlphaFold models of these proteins or their domains were fitted into the density. Atomic models were then built using Coot (*112*) and refined with Isolde (*113*) and REFMAC5 (*114*). Metal ions were assigned on the basis of coordinating residues and distances. Histidine modifications were built into the PP, PIP, and TBP.

### Ion assignment during model building

Zinc ions were assigned on the basis of the presence of coordinating histidine residues and the observed coordination geometry, octahedral in the TFs and pseudotetrahedral in the THP, which is predicted to function as a deacylase. Potassium ions, present in the buffer, were identified in the cryo-EM maps based on ligand-to-metal distances and coordination geometry consistent with long-wavelength x-ray data near the K^+^ absorption edge for the *Thermus thermophilus* ribosome (*115*). In this reference, K^+^ ions are predominantly coordinated by oxygen ligands and exhibit relatively long metal–ligand distances (>2.6 Å).

Both Mg^2+^ and Na^+^ ions were also present at high concentrations in the buffer. These cations are characterized by shorter ligand-to-metal distances (1.9 to 2.3 Å). However, the resolution of the HFTV1 cryo-EM maps does not permit modeling of coordinating water molecules, which would help to distinguish Mg^2+^ from Na^+^. Notably, Mg^2+^ ions typically complete an octahedral coordination sphere, whereas Na^+^ ions have a more variable and less ordered geometry. Given the previously reported importance of Mg^2+^ for virus stability in this system, we modeled all oxygen-coordinated ions with short ligand-to-metal distances as Mg^2+^. Statistics pertaining to image processing and atomic model building have been summarized in table S2.

## Supplementary Materials

### The PDF file includes:

Figs. S1 to S14 Tables S1 and S2 Flowcharts S1 and S2 Legends for movies S1 to S4 Legends for datasets S1 to S4

### Other Supplementary Material for this manuscript includes the following:

Movies S1 to S4

Datasets S1 to S4
