# Supplementary material for: Cryo-EM resolves the structure of the archaeal dsDNA virus HFTV1 from head to tail
Source: Sci Adv. 2025 Oct 3;11(40):eadx1178. doi: 10.1126/sciadv.adx1178 (PMC12494034; doi:10.1126/sciadv.adx1178)
Supplement: Supplementary file 1 — Figs. S1 to S14 Tables S1 and S2 Flowcharts S1 and S2 Legends for movies S1 to S4 Legends for datasets S1 to S4 [file sciadv.adx1178_sm.pdf]

Science Advances
AAAS

Supplementary Materials for

Cryo-EM resolves the structure of the archaeal dsDNA virus HFTV1 from head to tail

Daniel X. Zhang *et al.*

Corresponding author: Bertram Daum, b.daum2@exeter.ac.uk

Sci. Adv. **11**, eadx1178 (2025)
DOI: 10.1126/sciadv.adx1178

The PDF file includes:

Figs. S1 to S14
Tables S1 and S2
Flowcharts S1 and S2
Legends for movies S1 to S4
Legends for datasets S1 to S4

Other Supplementary Material for this manuscript includes the following:

Movies S1 to S4
Datasets S1 to S4

Figures S1-S14

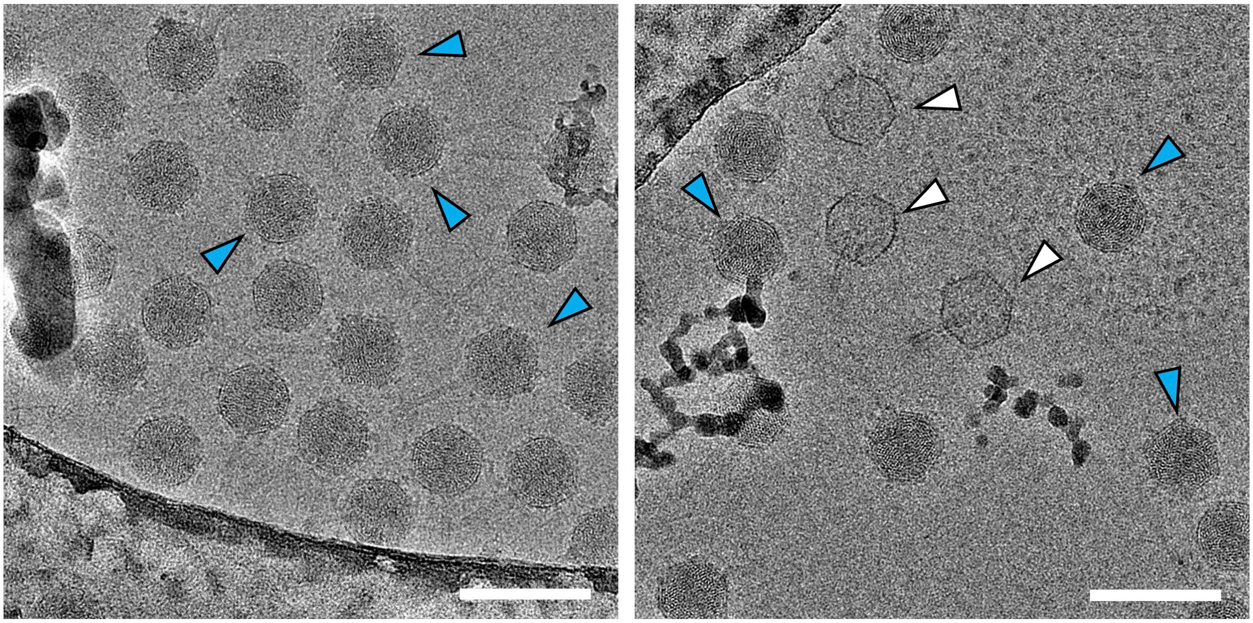

Fig. S1. Raw micrographs

Drift-corrected micrographs showing HFTV1 virions filled with DNA (blue arrowheads) and empty HFTV1 particles (white arrowheads). Scale bars, 100 nm.

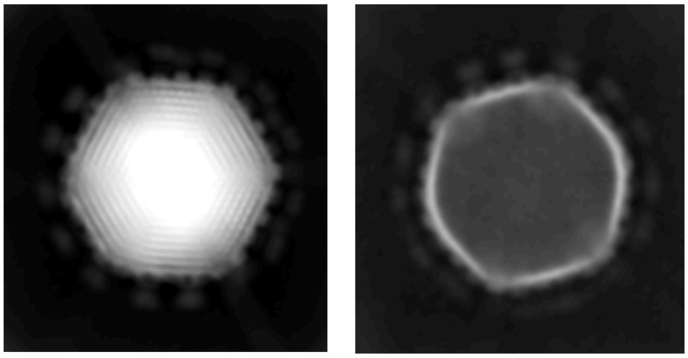

Fig. S2. 2D class averages of the HFTV1 capsid.
Example 2D classes showing the dsDNA-filled (left) and empty HFTV1 capsid (right).

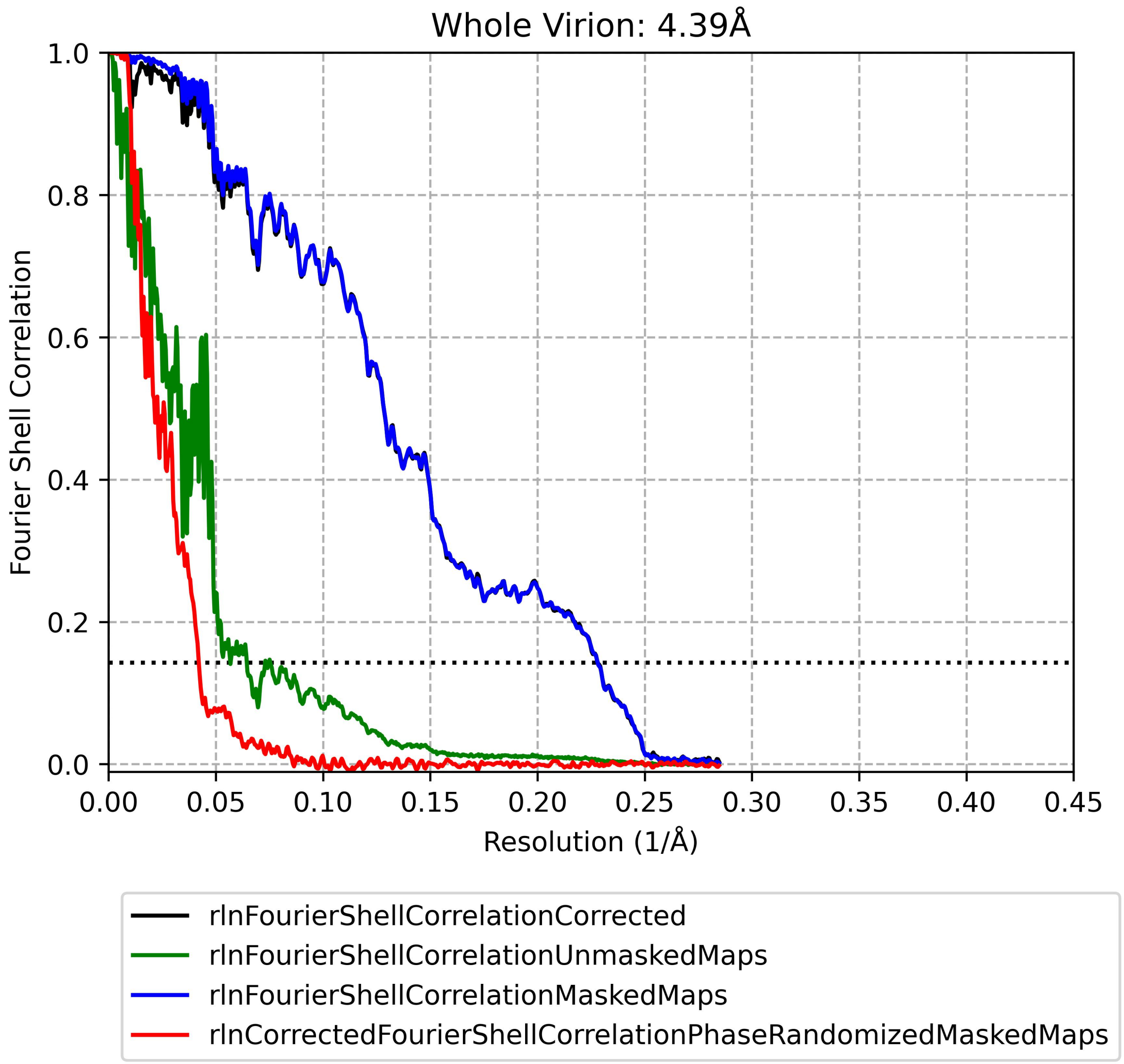

Fig. S3. Resolution estimation for C1 map of the whole virion

The resolution was estimated using Gold-standard Fourier shell correlation (FSC) at the 0.143 criterion.

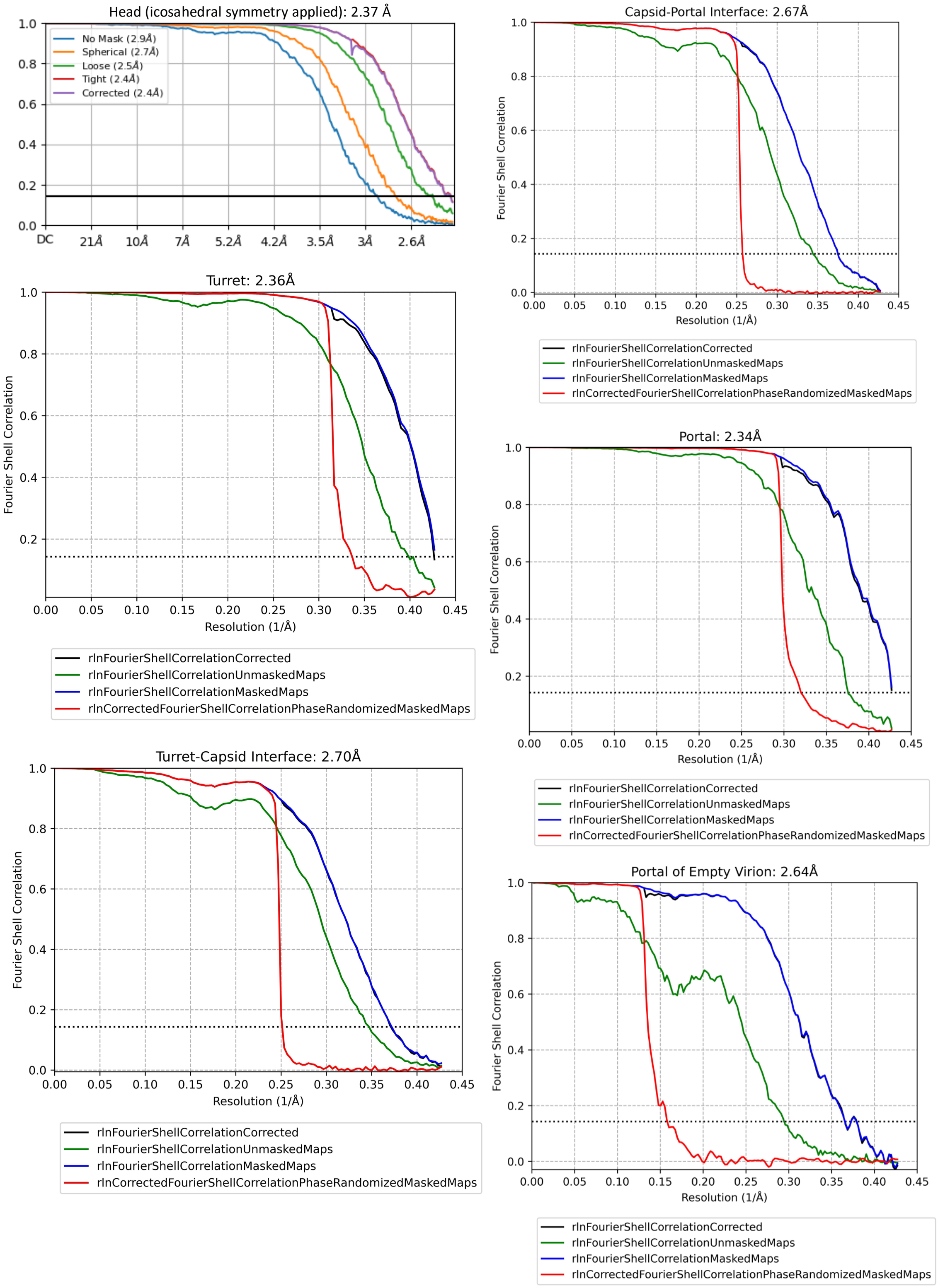

Fig. S4. Resolution estimation for different regions associated with the head of HFTV1.
The resolution was estimated using Gold-standard Fourier shell correlation (FSC) at the 0.143 criterion.

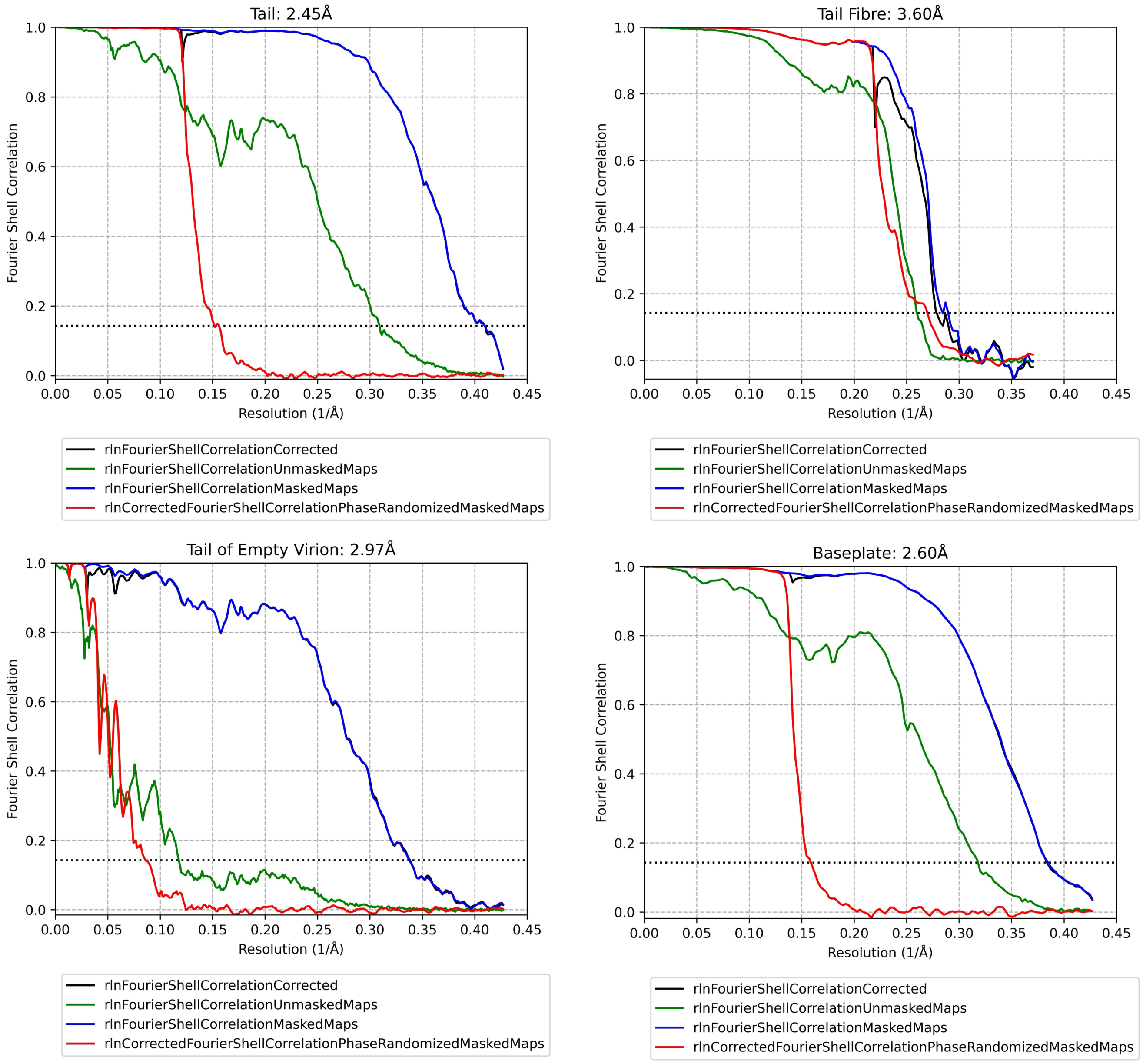

Fig. S5. Resolution estimation for different regions of the tail and base plate of HFTV1.
The resolution was estimated using Gold Standard Fourier Shell Corelation (FSC) at the 0.143 criterion.

A

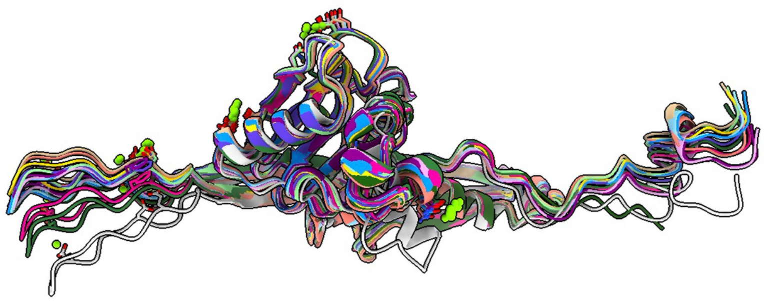

B

```
gp19 (genome)      MLMEAALPGSDVSAREVAKVWPGAKKGDYSFLQGNQSRSLEAEMTRTARA    50
gp19 (structure)   --------------------------------------------------     0


gp19 (genome)      EAGTDRHRALKDYAVDADNLPKTLSAGSKHLTEDGDVIEARLDDAIPRML   100
gp19 (structure)   --------------------------------------------------     0


gp19 (genome)      FAASDPEYVDTLFREQLLEVVMEGRELRKVAREASNVINANTRVGDVPIA   150
gp19 (structure)   FAASDPEYVDTLFREQLLEVVMEGRELRKVAREASNVINANTRVGDVPIA    50
                   **************************************************

gp19 (genome)      SDEEFARPTGQGAEIRDDGETYTTVAWNATKLTEGSRVTDEMRDQAMVDL   200
gp19 (structure)   SDEEFARPTGQGAEIRDDGETYTTVAWNATKLTEGSRVTDEMRDQAMVDL   100
                   **************************************************

gp19 (genome)      IERNIQRVGASLENGINRVFLTELVDNAQNNHDTAGSNQGYQALNSAVGE   250
gp19 (structure)   IERNIQRVGASLENGINRVFLTELVDNAQNNHDTAGSNQGYQALNSAVGE   150
                   **************************************************

gp19 (genome)      VDKDDFRPDTYVTHPDYRTQLFNDTNLAYANRAGTNEVLRNREDAPIVGD   300
gp19 (structure)   VDKDDFRPDTYVTHPDYRTQLFNDTNLAYANRAGTNEVLRNREDAPIVGD   200
                   **************************************************

gp19 (genome)      IAGLDMHAAMSSATYDDGTDIGWSGGSETWGFSSDGDKGAVVYDRDNIHT   350
gp19 (structure)   IAGLDMHAAMSSATYDDGTDIGWSGGSETWGFSSDGDKGAVVYDRDNIHT   250
                   **************************************************

gp19 (genome)      ILYAPNGQDVEIKDYEDPIRDITGVNGRLHVDCQYSQGRSSATVQY       396
gp19 (structure)   ILYAPNGQDVEIKDYEDPIRDITGVNGRLHVDCQYSQGRSSATVQY       296
                   **********************************************
```

Fig. S6. Conformational space and sequence of HFTV1-MCP.
A, 13 conformers of HFTV1-MCP superimposed. **B**, alignment between the HFTV1 MCP sequence based on the annotated genome (NC_062739.1) and that determined from the structure.

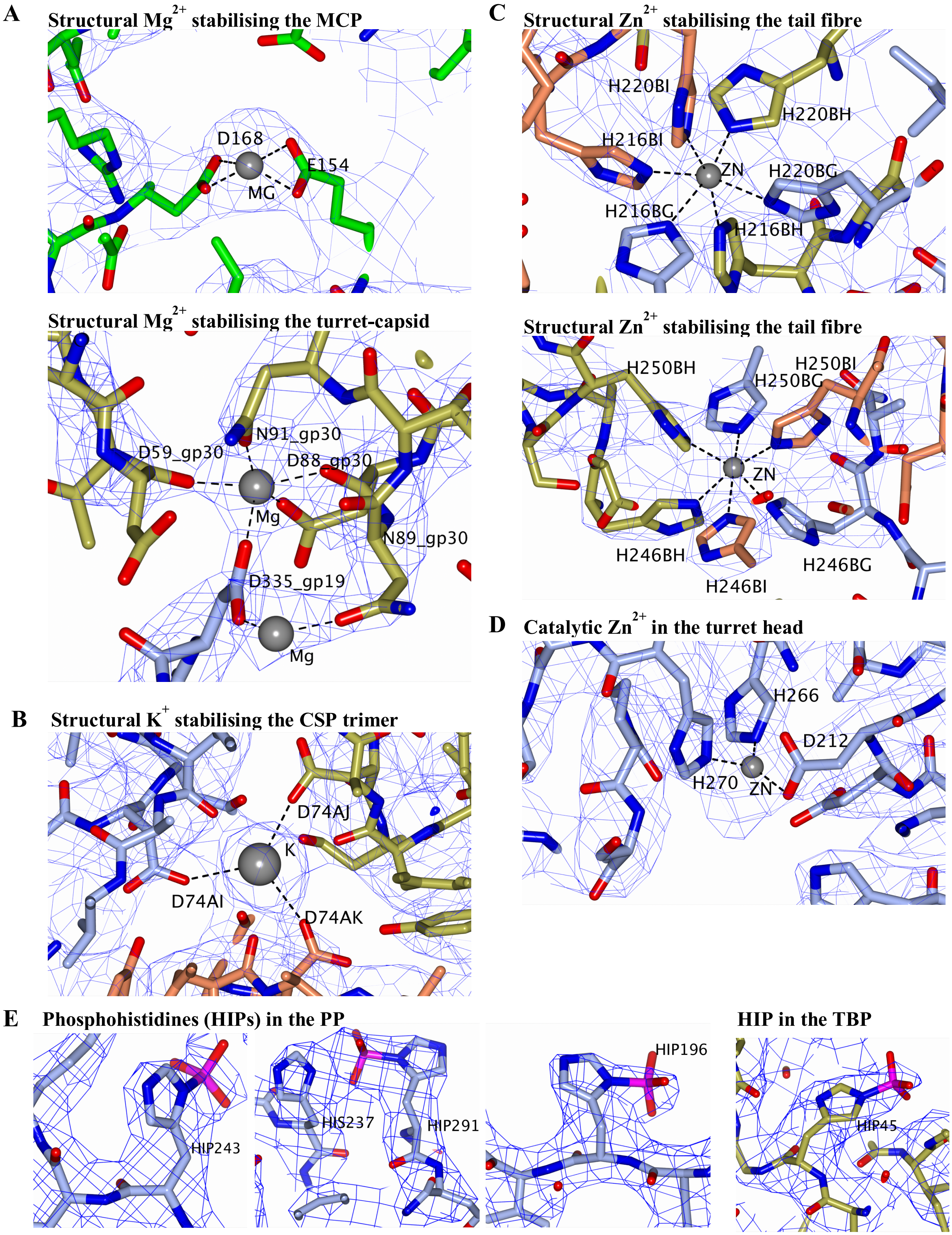

Fig. S7. Examples of metal ion coordination and phosphohistidine modifications in HFTV1.

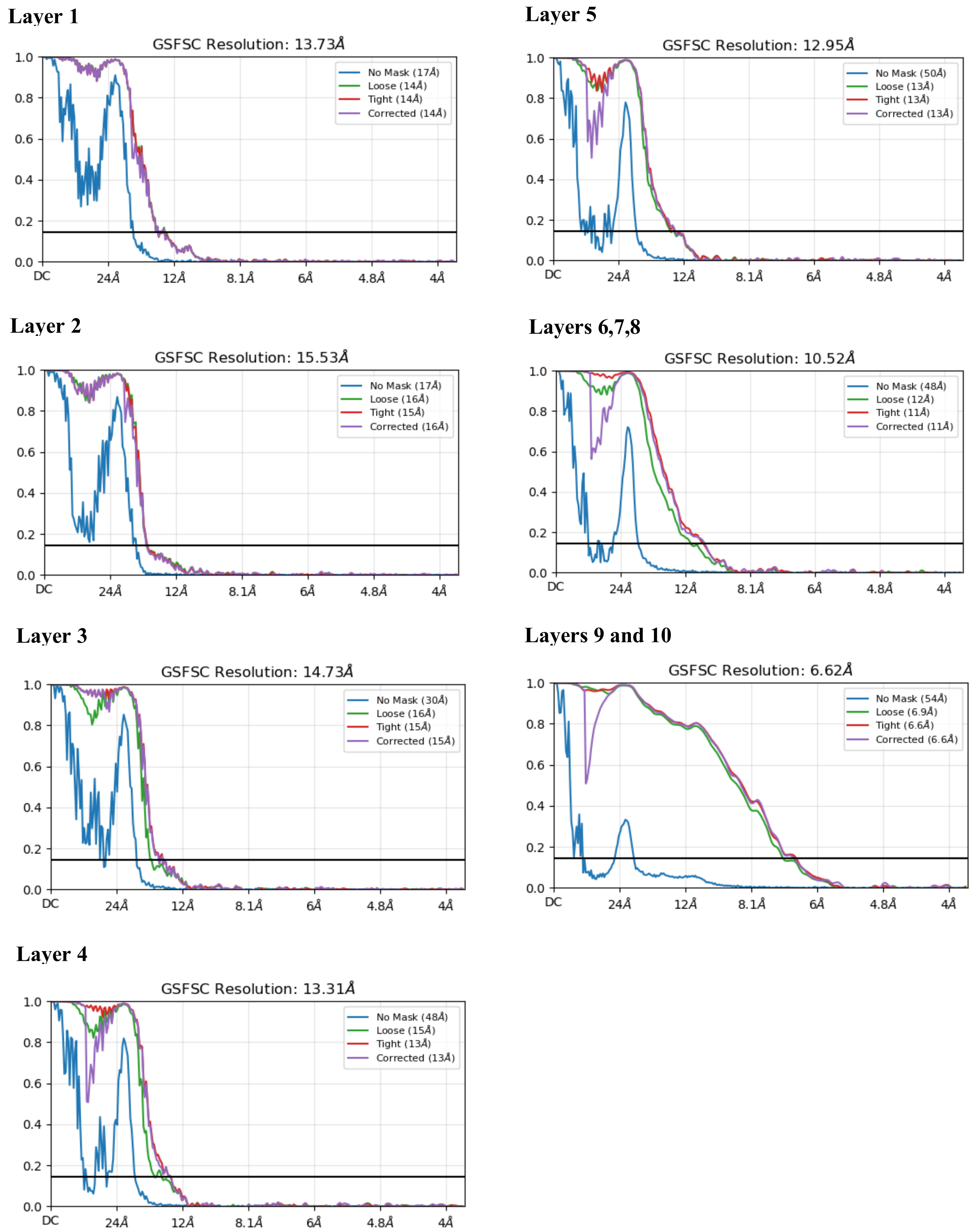

Fig. S8. Resolution estimation for the dsDNA layers within the capsid of HFTV1
The resolution was estimated using Gold-standard Fourier shell correlation (FSC) at the 0.143 criterion.

```
gp30 (genome)      --------------------------------------------------    0
gp30 (structure)   MTDTIVNVQGSFFSASASGVADTESLLIDPQDAKFGAIEIHNIAHGGSVD   50


gp30 (genome)      MELLTSSDDTELVEDAAVTLDSFTGEGISQGNQIEASDNTNTYIRITNTS   50
gp30 (structure)   VELLTSSDDTELVEDAAVTLDSFTGEGISQGNQIEASDNTNTYIRITNTS  100
                   .*************************************************

gp30 (genome)      GGAIDIIATGREVSQ                                      65
gp30 (structure)   GGAIDIIATGREVSQ                                     115
                   ***************
```

Fig. S9. Turret base protein (TBP; gp30) genome annotation vs. structure

Alignment between the HFTV1 turret base protein (TBP; gp30) sequence based on the genome sequence (NC_062739.1) and the TBP sequence determined from the structure

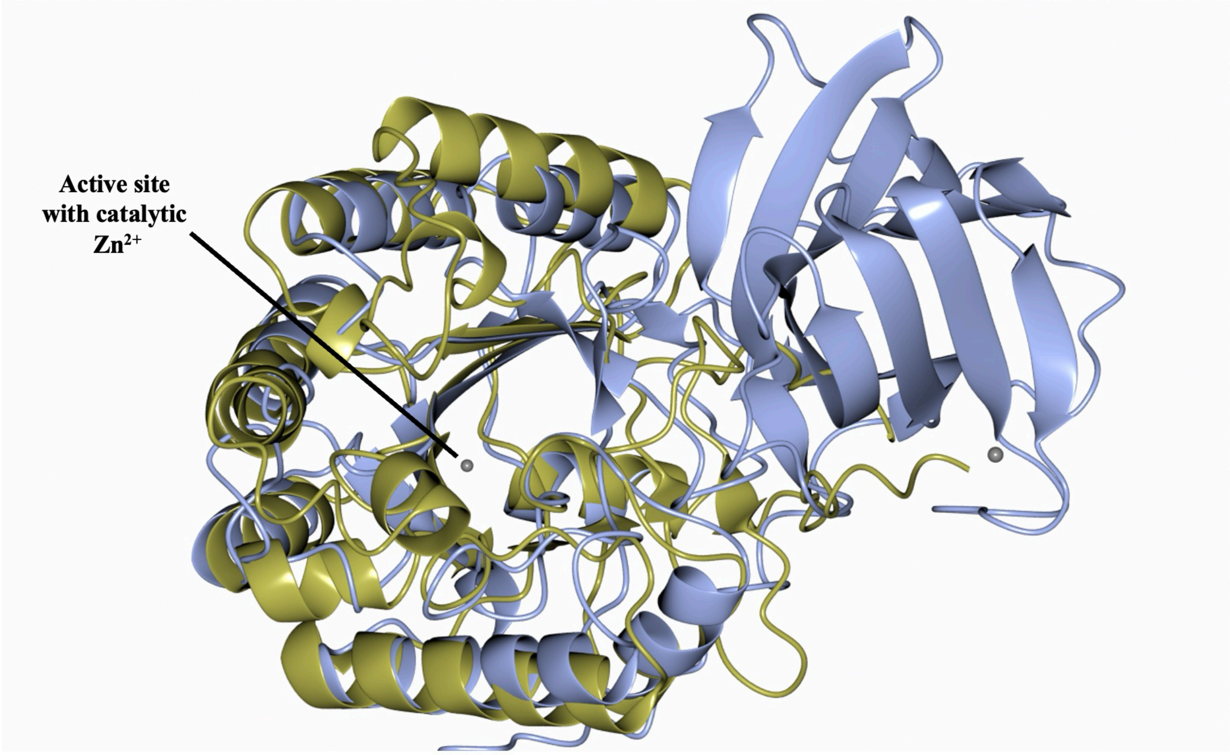

Fig. S10. The HFTV1 turret head protein (THP; gp31) contains a putative polysaccharide deacetylase domain. Superimposition of the structure of the polysaccharide deacetylase from *Mycobacterium smegmatis* (PDB-3RXZ; yellow) and the HFTV1 turret head protein (THP, blue). Protein structures are depicted in ribbon representation. Coordinated ions are shown as spheres.

```
gp21 (genome)      --------MRPMDRDWHQERARAREQAYSSDLTSQFSESEIVKYELDTAQ    42
gp21 (structure)   MQLRRSPGMRPMDRDWXQERARAREQAYSSDLTSQFSESEIVKYELDTAQ    50
                           ********:*********************************

gp21 (genome)      IDGSDNPRTYIWNRTIDLFGMNGTDVRELRNR                      74
gp21 (structure)   IDGSDNPRTYIWNRTIDLFGMNGTDVRELRNR                      82
                   ********************************
```

Fig. S11. Genome annotation vs structure of PIP (gp21)
Alignment between the sequence of the portal interface protein (PIP; gp21) as it was annotated in the genome (NC_062739.1), with that determined from the structure.

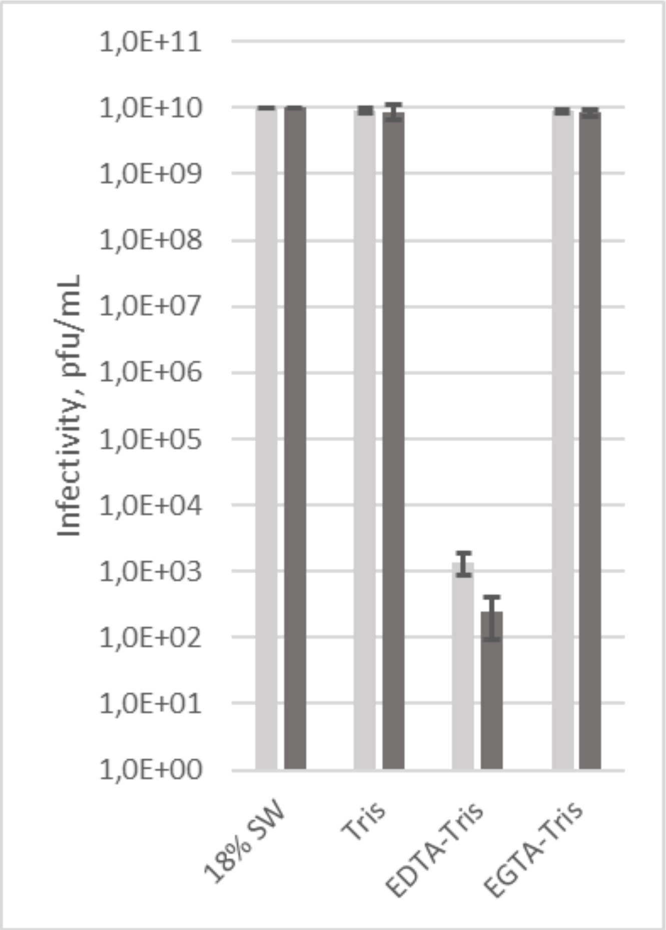

Fig. S12. EDTA treatment reduces the infectivity of HFTV1
Infectivity of HFTV1 in 18% SW (positive control; 2.47 M NaCl, 89 mM $MgCl_2$, 72 mM $MgSO_4$, 56 mM KCl, 3 mM $CaCl_2$, 50 mM Tris-HCl pH 7.2); Tris (50 mM Tris-HCl pH 7.2), EDTA-Tris (10 mM EDTA, 50 mM Tris-HCl pH 7.2); and EGTA-Tris buffer (10 mM EGTA, pH 7.9, 50 mM Tris-HCl pH 7.2). Infectivity was determined after 2 h (light grey) or 24 h (dark grey) incubation by plaque assays. The error bars show the standard deviation (n=3).

A

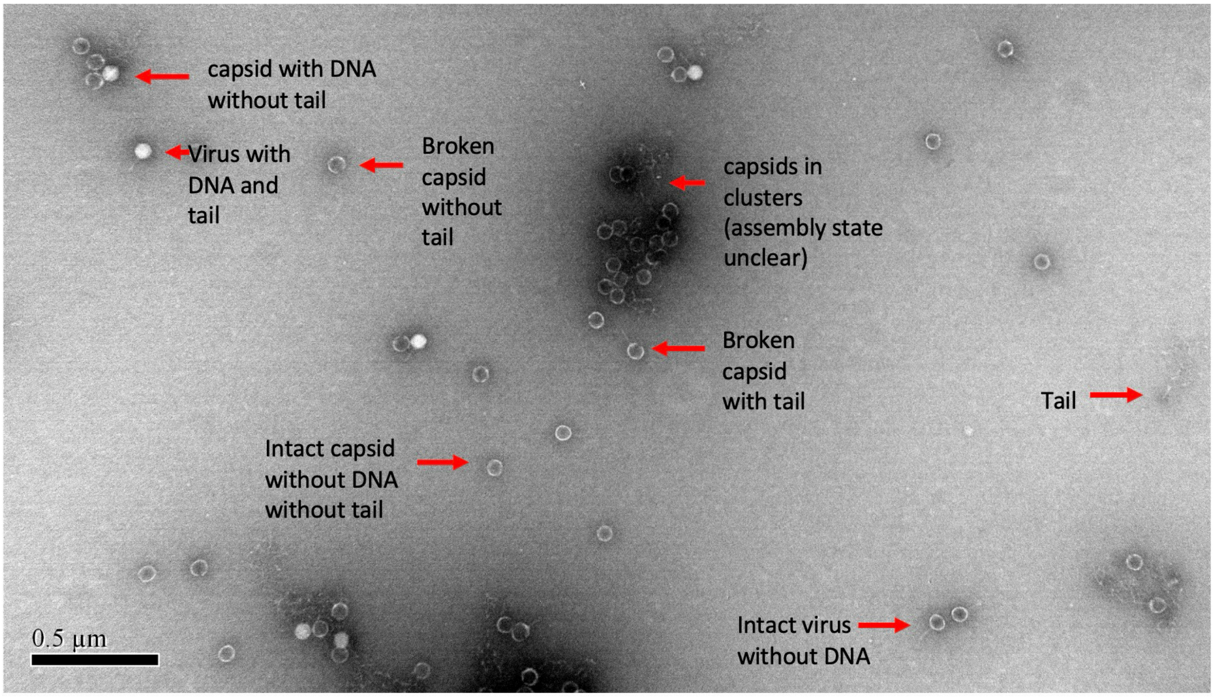

B

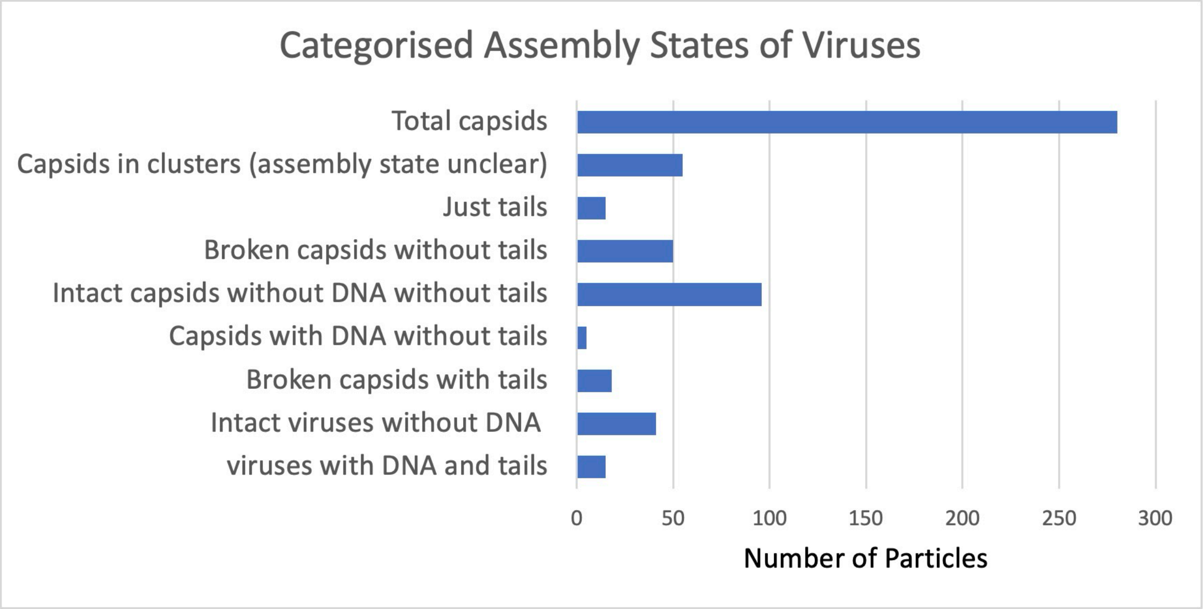

Fig. S13. EDTA treatment disintegrates HFTV1
A, negative stain micrograph of HFTV1 after Mg^{2+} depletion (see Supplementary Figure 12, EDTA-Tris sample). Different HFTV1 virion subcomplexes are indicated. **B**, bar chart showing the distribution of different virion subcomplexes over 100 micrographs.

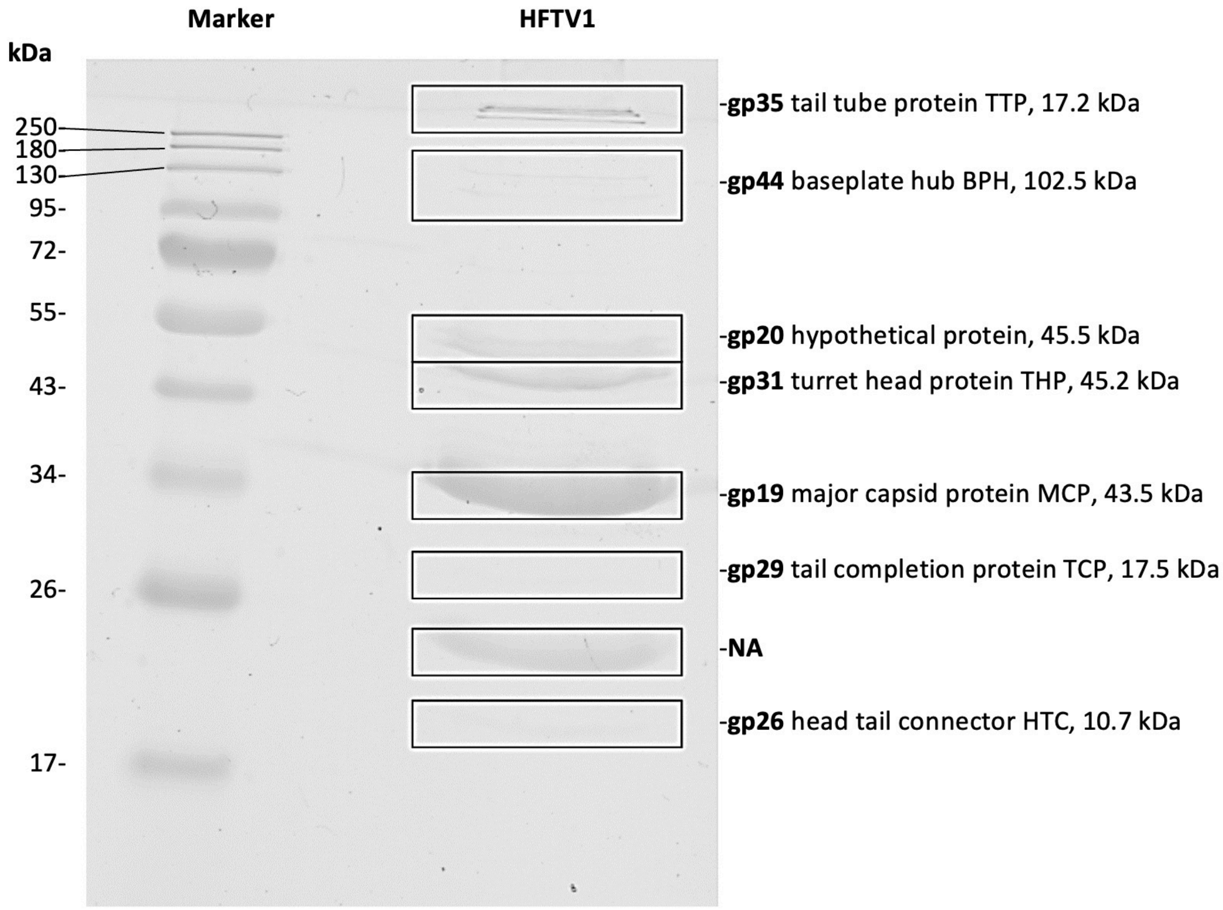

Fig. S14. SDS-PAGE analysis of proteins from purified HFTV1 particles.
The proteins of the purified HFTV1 virion were separated by SDS-PAGE (stained with Coomassie blue) and identified by HPLC-MS/MS.

Supplementary Table 1 - Cryo-EM data collection statistics

	Dataset 1	Dataset 2
	EMPIAR-12533	EMPIAR-12533
**Data collection and processing**		
Microscope	Thermo Fisher Scientific (TFS) Titan Krios	
Voltage (kV)	300	
Camera	Gatan K3	TFS Falcon 4i with Selectris X
Electron exposure ($e^{-}/Å^{2}$)	54.6	50.0
Frames collected per movie (no.)	52	45
Defocus range (μm)	-0.8 to -2.0	
Magnification	64 k	105 k
Pixel size (Å)	1.35 (0.675 super-resolution)	1.171
Movies collected (no.)	10,236	19,717
Initial particle images (no.)	147,736	80,058

Supplementary Table 2 - Cryo-EM data processing, refinement, and validation statistics

	Turret	Turret-capsid interface	Portal	Portal of empty virion	Capsid-portal interface	Capsid	Tail	Tail of empty virion	Baseplate	Tail fibre
EMDB accession code	18550	18559	18599	18633	18642	51530	51866	50521	51883	51915
PDB accession code	8QPG	8QPQ	8QQN	8QSI	8QSY	9GS0	9H4P	9FKB	9H7V	9H5B
**Data collection and processing**										
Final particle images (no.)										
Dataset 1	—	—	122,808	4,209	115,326	—	84,928	3,270	95,147	251,945
Dataset 2	267,124	102,012	42,390	3,372	42,062	41,875	31,952	3,310	33,248	84,509
Symmetry applied	C3	C1	C12	C12	C1	I3	C3	C3	C1	C3
Map resolution (Å)	2.36	2.70	2.34	2.64	2.67	2.37	2.45	2.97	2.60	3.60
FSC threshold					0.143					
Map resolution range (Å)	2.34-3.05	2.44-3.92	2.34-2.90	2.56-3.75	2.43-3.97	2.37-5.2	2.34-3.83	2.8-41.0	2.4-11.8	3.38-4.01
**Refinement**										
Initial model used	-	-	-	-	-	-	-	-	-	-
Map/Model resolution (Å)	2.4	2.7	2.37	2.9	2.7	2.9	3.5	3.2	3.1	3.7
FSC threshold						0.5				
Map sharpening *B* factor (Å^2)	0	0	0	0	0	0	0	0	0	0
Model composition										
Non-hydrogen atoms	14,550	28,263	50,328	50,784	137,239	27,880	179,448	117,625	54,698	9,801
Protein residues	1,920	3,683	6,432	6,492	17,733	3,708	23,358	15,238	7,347	1,342
Ligands (glycans)	24	53	12	12	200	51	15	12	62	11
B factors (Å^2)										
Protein	68.9	92.6	82.0	74.7	65.6	157.8	47.5	87.1	43.6	119.4
Ligand	81.8	91.8	79.7	67.0	86.4	7.5	9.2	230.7	30.1	117.8
R.m.s. deviations										
Bond lengths (Å)	0.009	0.009	0.027	0.027	0.018	0.014	0.017	0.016	0.007	0.007
Bond angles (°)	1.596	1.703	1.703	1.696	1.598	1.591	1.568	1.999	1.439	1.523
Validation										
MolProbity score	1.03	1.01	0.68	0.95	0.85	1.05	0.77	0.87	1.01	1.81
Clashscore	1.74	0.93	0.46	0.42	0.20	1.00	0.21	0.68	0.56	2.92

Poor rotamers (%)	0.31	0.82	0.83	0.39	0.94	1.29	0.99	0.88	0.99	1.72
Ramachandran plot										
Favored (%)	97.51	96.48	97.94	95.67	96.06	97.03	96.91	97.15	95.41	90.03
Allowed (%)	2.49	3.52	2.06	4.33	3.94	2.97	3.08	2.85	4.66	9.97
Disallowed (%)	0.0	0.0	0.0	0.0	0.0	0.0	0.0	0.0	0.04	0.0

CryoEM Flowchart 1
Image processing flowchart for the HFTV1 capsid

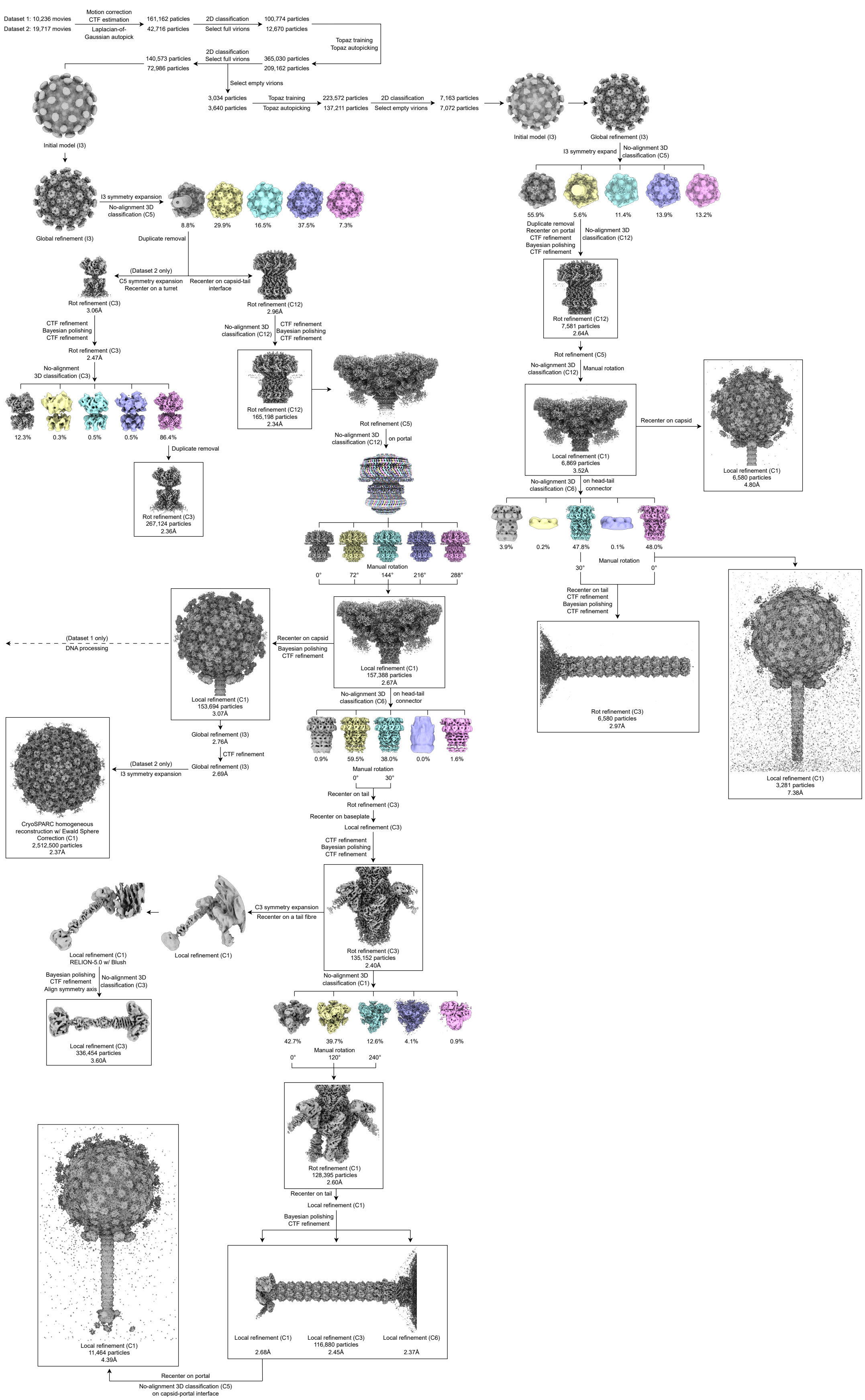

CryoEM Flowchart 2

Image processing flowchart for spooled dsDNA within the head of the HFTV1 capsid

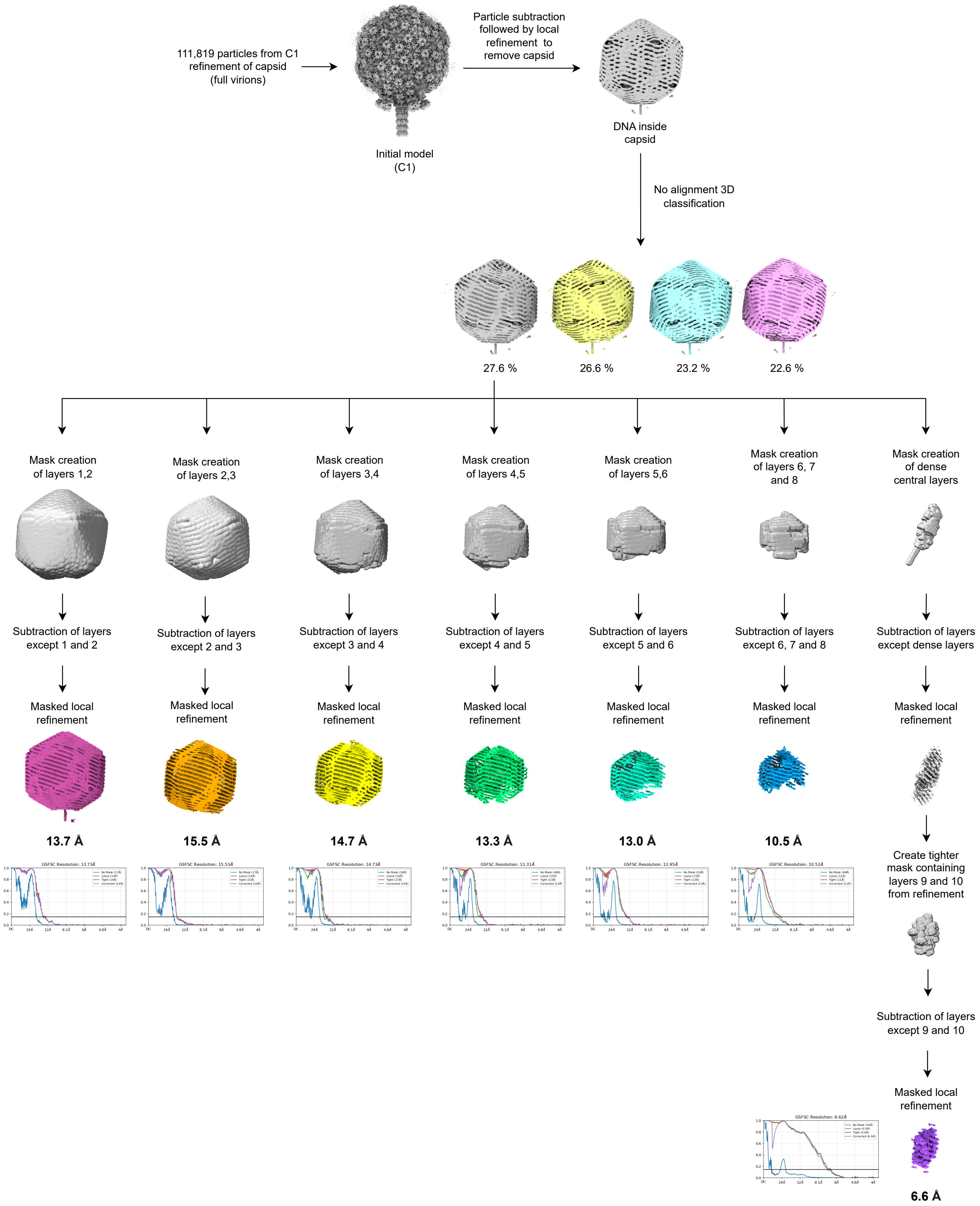

Captions for other Supplementary Materials associated with this manuscript:

Movie S1. (separate file)
Structure of HFTV1

Movie S2. (separate file)
Conformational space of HFTV1-MCP

Movie S3. (separate file)
Different conformations that can be adopted by the baseplate hub (BPH) trimer

Movie S4. (separate file)
Flexibility of the tail fibres

Data S1. (separate file) Mass spectrometry analysis of the infectious virions

Data S2. (separate file) HHpred results

Data S3. (separate file) HHpred, ArCOG and InterPro annotations for the HFTV1 genome

Data S4. (separate file)
DALI analysis
